# Epigenetic regulation in development: is the mouse a good model for the human?

**DOI:** 10.1093/humupd/dmy021

**Published:** 2018-07-10

**Authors:** Courtney W Hanna, Hannah Demond, Gavin Kelsey

**Affiliations:** 1 Epigenetics programme, Babraham Institute, Cambridge, UK; 2 Centre for Trophoblast Research, University of Cambridge, Cambridge, UK

**Keywords:** DNA methylation, imprinting, development, epigenetics, oocyte, embryo, sperm, chromatin, histones

## Abstract

**BACKGROUND:**

Over the past few years, advances in molecular technologies have allowed unprecedented mapping of epigenetic modifications in gametes and during early embryonic development. This work is allowing a detailed genomic analysis, which for the first time can answer long-standing questions about epigenetic regulation and reprogramming, and highlights differences between mouse and human, the implications of which are only beginning to be explored.

**OBJECTIVE AND RATIONALE:**

In this review, we summarise new low-cell molecular methods enabling the interrogation of epigenetic information in gametes and early embryos, the mechanistic insights these have provided, and contrast the findings in mouse and human.

**SEARCH METHODS:**

Relevant studies were identified by PubMed search.

**OUTCOMES:**

We discuss the levels of epigenetic regulation, from DNA modifications to chromatin organisation, during mouse gametogenesis, fertilisation and pre- and post-implantation development. The recently characterised features of the oocyte epigenome highlight its exceptionally unique regulatory landscape. The chromatin organisation and epigenetic landscape of both gametic genomes are rapidly reprogrammed after fertilisation. This extensive epigenetic remodelling is necessary for zygotic genome activation, but the mechanistic link remains unclear. While the vast majority of epigenetic information from the gametes is erased in pre-implantation development, new insights suggest that repressive histone modifications from the oocyte may mediate a novel mechanism of imprinting. To date, the characterisation of epigenetics in human development has been almost exclusively limited to DNA methylation profiling; these data reinforce that the global dynamics are conserved between mouse and human. However, as we look closer, it is becoming apparent that the mechanisms regulating these dynamics are distinct. These early findings emphasise the importance of investigations of fundamental epigenetic mechanisms in both mouse and humans.

**WIDER IMPLICATIONS:**

Failures in epigenetic regulation have been implicated in human disease and infertility. With increasing maternal age and use of reproductive technologies in countries all over the world, it is becoming ever more important to understand the necessary processes required to establish a developmentally competent embryo. Furthermore, it is essential to evaluate the extent to which these epigenetic patterns are sensitive to such technologies and other adverse environmental exposures.

## Introduction

**Figure 1:**
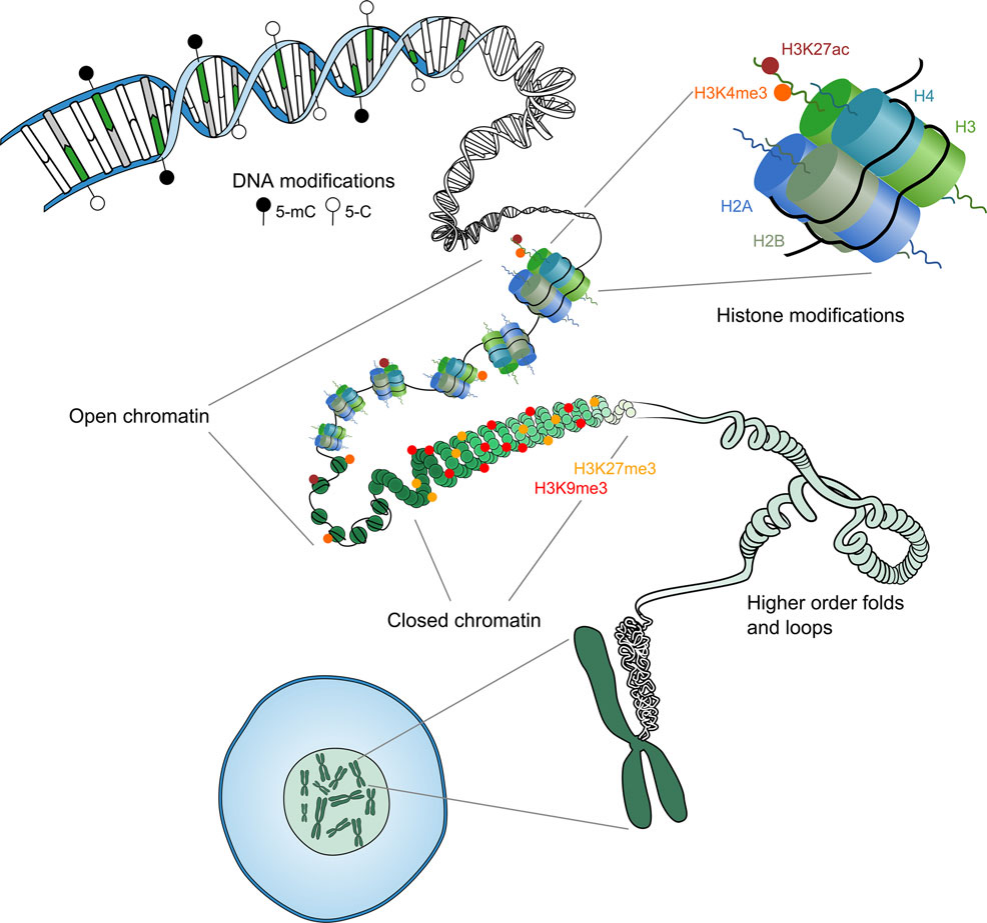
Levels of epigenetic regulation. The DNA sequence can be methylated at cytosine residues in a CpG context, termed DNA methylation. DNA is wrapped around the histone octamer to form the nucleosome. Variants and post-translational modifications of these histone proteins form another layer of epigenetic regulation. The state of these epigenetic modifications together determines whether the chromatin will be organised in an accessible ‘open’ or an inaccessible ‘closed’ state. Higher order folds and loops organise the chromatin into active and inactive compartments.

All cell types of an organism contain identical genetic information and yet are distinct in function and characteristics. Instructive epigenetic marks are key to this developmental conundrum. Epigenetic marks include modifications to the DNA or its associated proteins, which enable regulation of gene expression in a cell type-specific manner (Fig. 1). Among the most well-characterised epigenetic modifications is DNA methylation, but the various additional layers of epigenetic information may represent more dynamic and responsive features of this regulation landscape. DNA is wrapped around an octamer of histone proteins (a nucleosome) enabling its compaction and organisation in the nucleus. Numerous post-translational modifications and/or variants of these histone proteins can facilitate the packaging of chromatin into accessible or inaccessible states and, consequently, regions of active or repressed gene expression, respectively.

The field of developmental biology has long studied the intriguing nature of how two fully differentiated and very distinct cells, the sperm and the oocyte, can come together to create a totipotent embryo. Genetic studies in mice have firmly established that epigenetic regulation is key to the acquisition of totipotency during this transition. Early studies using molecular approaches and immunofluorescence showed that widespread epigenetic reprogramming accompanies both germ cell and embryonic development (Santos *et al.*, 2002; Seisenberger *et al.*, 2012). However, limitations in obtaining large numbers of cells, specifically in oogenesis and early embryogenesis, has restricted the detailed molecular investigation in these cells, until recently. Advances in low-input and single-cell sequencing methods have not only improved our understanding of these developmental windows, but the data have also led to new questions and challenged existing dogmas/hypotheses. In this review, we summarise the current knowledge of epigenetic dynamics in development, from DNA methylation to chromosome organisation, specifically during spermatogenesis, oogenesis, pre-implantation development and early lineage specification. We will discuss how the mechanistic insights established in mice may be relevant for human development and reflect on known differences between the two systems. In this review, we focus particularly on the recent developments in *in-vivo* studies.

## Recent advances in epigenetic profiling technologies

Next generation sequencing based approaches have revolutionised our ability to profile epigenetic information and all layers of the epigenome can now be interrogated by these methods. Until relatively recently, these technologies have required millions of cells to obtain high resolution genomic maps, but advances in capturing and amplifying smaller and smaller amounts of material have allowed them to be scaled down to require only minimal numbers of cells (Table I).

**Table I:** Low-input and single cell methods available for assaying epigenetic modifications.

Epigenetic layer	Assay	Low-cell protocol	Single-cell protocol
DNA methylation	Post-bisulfite adaptor tagging (PBAT)	400 cells (Miura *et al.*, 2012)	Yes (Smallwood *et al.*, 2014)
	Reduced representation bisulfite sequencing (RRBS)	75–1000 cells (Smallwood and Kelsey, 2012)	Yes (Guo *et al.*, 2013)
Histone modifications	Chromatin immunoprecipitation (ChIP)-seq	400–1000 cells (Brind’Amour *et al*, 2015, Zhang *et al.*, 2016, Dahl *et al.*, 2016, Hanna *et al.*, 2018)	Yes* (Rotem *et al.*, 2015)
	Cleavage under targets and release using nuclease (CUT&RUN)	100 cells (Skene *et al.*, 2018)	Not available
Chromatin accessibility	Assay for transposase accessible chromatin (ATAC)-seq	20–100 cells (Wu *et al.*, 2016, Wu *et al.*, 2018)	Yes (Buenrostro *et al.*, 2015; Cusanovich *et al.*, 2015)
	DNase-seq	100–200 cells (Lu *et al.*, 2016)	Yes (Jin *et al.*, 2015)
DNA methylation and chromatin accessibility	Nucleosome occupancy and methylome (NOMe)-seq	Not available	Yes (Pott, 2017, Guo *et al.*, 2017a, Clark *et al.*, 2018)
Higher order organisation	Hi-C	500 cells (Du *et al.*, 2017)	Yes (Nagano *et al.*, 2013)

*Only applied using thousands of cells.

DNA methylation can be studied with greatest resolution and precision by bisulphite conversion followed by sequencing (Cokus *et al.*, 2008). Bisulphite treatment converts the DNA base cytosine to uracil, but only when the cytosine is unmethylated. In this manner, methylated and unmethylated cytosines can be distinguished by sequencing. Bisulphite sequencing initially required large amounts of starting material because the bisulphite conversion reaction leads to DNA breaks and loss of material. This problem has been overcome by refinements in methods such as post bisulphite-adaptor tagging (PBAT) and reduced representation bisulphite (RRBS) sequencing, which allow the interrogation of DNA methylation in just 100–200 cells or even on a single-cell level (Miura *et al.*, 2012; Smallwood and Kelsey, 2012; Guo *et al.*, 2013; Smallwood *et al.*, 2014). Methods independent of bisulphite chemistry may provide alternatives that circumvent the loss of material inherent in bisulphite treatment (Boers *et al.*, 2018). A variety of approaches have also been developed to map oxidation derivatives of 5-methylcytosine, some at the single-cell level, but they typically lack the sensitivity or absolute quantification of bisulphite sequencing (Kelsey *et al.*, 2017).

Histone proteins can be post-translationally modified at numerous amino acid residues in the protruding N-terminal tail or core domain (Zhao and Garcia, 2015); these predominantly include methylation, acetylation, phosphorylation and ubiquitination. Using antibodies, the abundance and nuclear distribution of these modification states have been studied by immunofluorescence and Western blots. Determining their genomic occupancy depends upon using antibodies to precipitate chromatin fragments (chromatin immunoprecipitation, ChIP) followed by purification of the associated DNA. In 2006, next-generation sequencing was applied for the first time to obtain genome-wide maps of histone modifications, in a method termed ChIP-seq (Barski *et al.*, 2007; Mikkelsen *et al.*, 2007; Robertson *et al.*, 2007). This method is an enrichment-based approach that is strongly dependent on antibody efficiency and specificity. Only recently, ChIP-seq has been adapted for low-cell inputs of 500–1000 cells (Brind’Amour *et al.*, 2015; Dahl *et al.*, 2016; Zhang *et al.*, 2016; Hanna *et al.*, 2018), and single-cell approaches still require the processing of thousands of individual cells (Rotem *et al.*, 2015). A novel approach, termed cleavage under targets and release using nuclease (CUT&RUN), has been developed to allow the evaluation of histone modification patterns in as few as 100 cells (Skene *et al.*, 2018). CUT&RUN involves tethering a DNA-cutting enzyme to a histone-bound antibody, resulting in only targeted DNA-wrapped nucleosomes being released into solution for sequencing (Skene *et al.*, 2018).

Chromatin states can be analysed further by a variety of methods that use enzymes to isolate accessible from inaccessible regions of DNA. For example, the assay of transposase-accessible chromatin (ATAC-seq) employs the Tn5 transposase to integrate sequencing adapters into regions of accessible chromatin (Buenrostro *et al.*, 2013), while DNase-seq employs the DNase I enzyme to cleave these regions (Boyle *et al.*, 2008). Both methods have recently been adapted for single-cell and low-cell input (Buenrostro *et al.*, 2015; Cusanovich *et al.*, 2015; Jin *et al.*, 2015; Lu *et al.*, 2016; Wu *et al.*, 2016). An alternative assay, termed nucleosome occupancy and methylome (NOMe-seq), uses a unique non-enrichment-based approach to evaluate chromatin accessibility, by exploiting a bacterial methyltransferase (Kelly *et al.*, 2012). Accessible regions of DNA are demarked with GpC methylation, and therefore subsequent bisulphite sequencing not only provides information on DNA accessibility but additionally endogenous DNA methylation patterns. NOMe-seq has been successfully adapted to the single-cell level (Pott, 2017; Guo *et al.*, 2017a; Clark *et al.*, 2018).

On a larger scale, chromatin conformation capture (Hi-C) methods evaluate chromosome interactions from a defined loci or throughout the nucleus, using cross-linking to ligate regions of DNA that lie in close proximity to each other (Lieberman-Aiden *et al.*, 2009). The so-called topological associated domains (TADs) partition the genome into large self-interacting A (active) and B (silent) compartments. Hi-C sequencing can also be conducted on a single-cell level (Nagano *et al.*, 2013), but at rather limited resolution. At higher resolution, HiC-based methods can identify enhancer-promoter interactions, but this application is not yet possible in low numbers of cells.

## Epigenetic regulation of gene expression

**Figure 2:**
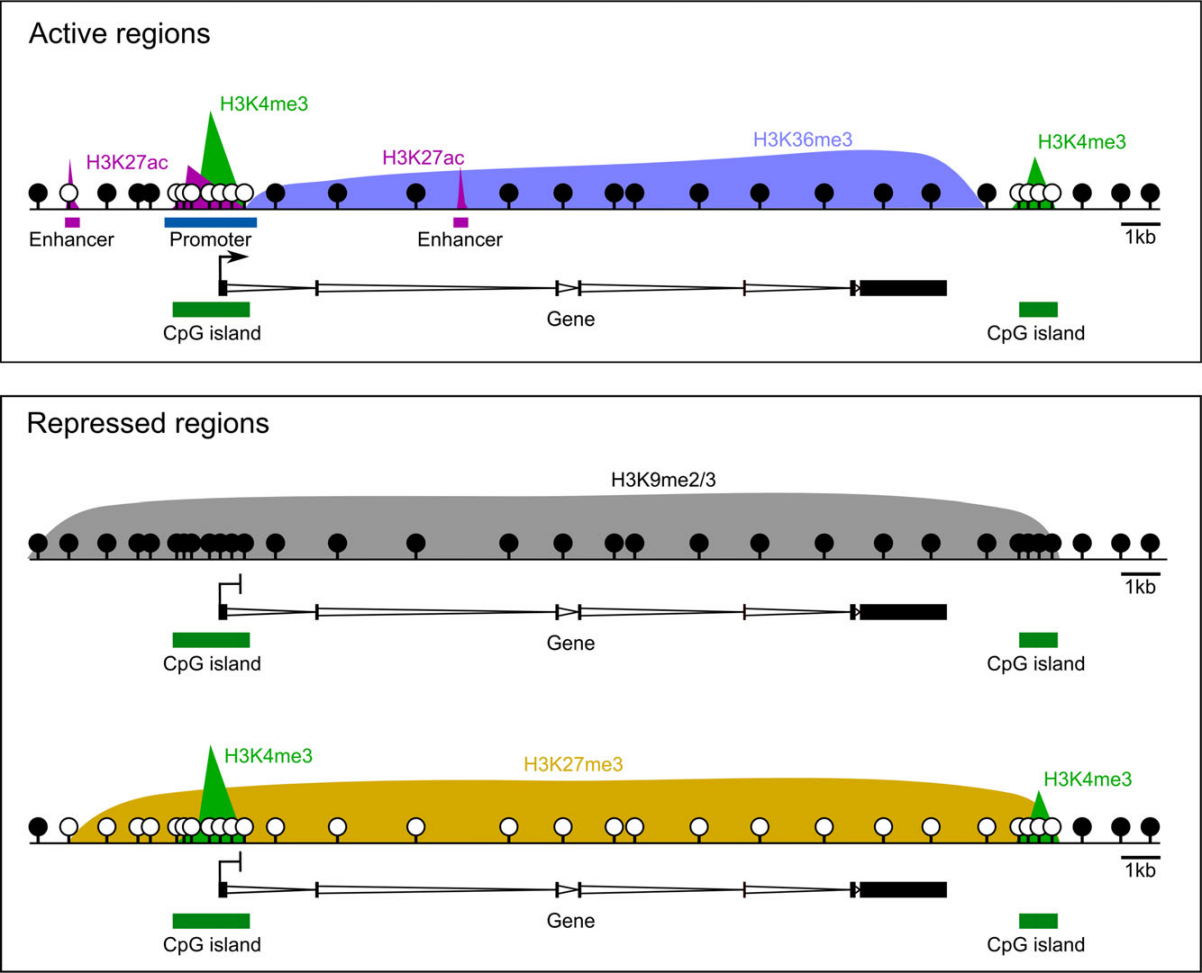
Canonical epigenetic patterns. H3K4me3 is associated with actively transcribed promoters, as well as CpG islands, irrespective of transcription. H3K27ac demarks active promoters and enhancers, while associated transcribed genes bodies are enriched for H3K36me3. Repressed regions of the genome are typically associated with either H3K9me2/3 or H3K27me3. DNA is generally highly methylated throughout the genome, with the exception of regulatory regions marked by H3K4me3 and/or H3K27ac, and H3K27me3- domains. Methylated CpGs are depicted as closed circles and unmethylated CpGs are open circles.

In differentiated cells, there are canonical patterns of epigenetic marks across genomic elements (Fig. 2). DNA methylation is generally high across gene bodies and inter-genic regions, with low or intermediate methylation observed almost exclusively at regulatory regions, such as promoters and enhancers. Histone marks, typified by histone H3 modifications, also show reproducible genomic patterns, some of which are correlated with gene expression. Active marks, such as histone 3 lysine 4 trimethylation (H3K4me3) and/or histone 3 lysine 27 acetylation (H3K27ac), are found at active promoters and/or enhancers, are negatively correlated with DNA methylation, and positively correlated with gene expression (Fig. 2) (Smith and Meissner, 2013). Repressive histone marks, such as H3K36me3 across transcribed gene bodies and H3K9me2 and/or H3K9me3, are strongly associated with DNA methylation and transcriptional silencing (Du *et al.*, 2015). While gene body H3K36me3 is positively correlated with transcription, paradoxically it is thought to function across gene bodies by repressing spurious, off-target transcription initiation (Neri *et al.*, 2017) and promoting acquisition of DNA methylation (Baubec *et al.*, 2015). Alternatively, while repressive H3K27me3 is associated with transcriptional silencing, it is predominantly localised with unmethylated DNA, suggesting it may be complementary mode of genomic silencing (Fig. 2) (Manzo *et al.*, 2017). While many other modifications of histone proteins have been reported (Zhao and Garcia, 2015), in this review we focus on the aforementioned well-characterised histone modifications.

DNA methylation is established and maintained by a protein family of five DNA methyltransferases (DNMTs). Among these, three *de-novo* DNMTs (DNMT3A, 3B and 3C) and a catalytically inactive co-factor (DNMT3L) are responsible for establishing cytosine methylation, usually in a CpG context (Okano *et al.*, 1999; Bourc’his *et al.*, 2001; Barau *et al.*, 2016). It is not fully apparent how DNA methylation is targeted to specific regions of the genome, but biochemical studies have shown that several domains on the DNMT proteins or their co-factors can interact with modified histone tails (Ooi *et al.*, 2007; Dhayalan *et al.*, 2010). During cell replication, DNMT1 is localised to hemi-methylated DNA at the replication fork by the co-factor UHRF1 (Bostick *et al.*, 2007; Sharif *et al.*, 2007), where it faithfully copies CpG methylation patterns to the newly replicated DNA strand (Li *et al.*, 1992). Once established, DNA methylation can be repressive for transcription either by impairing the binding of transcription factors or through the activity of methyl-binding proteins (Hendrich and Bird, 1998; Domcke *et al.*, 2015). Classic examples of the repressive role for DNA methylation are X-chromosome inactivation in females and imprinted gene regulation, where one parental allele is silenced through the inheritance of differential germline methylation (Jones, 2012). Methylated cytosine can be oxidised to the derivatives 5-hydroxymethylcytosine, 5-formylcytosine and 5-carboxylcytosine through the action of Ten-Eleven Translocation (TET) proteins, but whether these derivatives function as epigenetic marks in a manner similar to 5-methylcytosine is not clear (Wu and Zhang, 2017).

The modifications of histone tails are dynamically regulated by so-called ‘writers’ and ‘erasers’, and once established can be bound by ‘readers’ (Cheng, 2014; Rothbart and Strahl, 2014; Torres and Fujimori, 2015; Allis and Jenuwein, 2016). There is an ever-growing list of proteins that can modulate and/or bind histones (http://weram.biocuckoo.org/), suggesting that the complexity of this system is extensive. In general terms, active and repressive histone marks, through their respective readers, can enable the immediately surrounding chromatin to be packaged in an open (accessible) or closed (inaccessible) conformation, respectively (Zhang *et al.*, 2015). Regions of open or closed chromatin are organised into self-interacting compartments, termed TADs, which are on average ~1 Mb in size (Dixon *et al.*, 2012). Within the nucleus, TADs of similar chromatin conformation are more likely to organise together into active and inactive (A and B) compartments (Lieberman-Aiden *et al.*, 2009). This supports the notion that there is coordinate regulation of transcriptional activity through the 3D organisation of DNA within the nucleus.

Ongoing work in model systems including, but not limited to, the mouse is building our understanding of the interplay between these epigenetic layers and how they coordinate genomic regulation. One way to evaluate these relationships is to study their dynamics during developmental reprogramming and lineage specification, an area of research that has rapidly advanced in the past few years.

## Mechanistic insights from mouse models

### Gametogenesis

**Figure 3:**
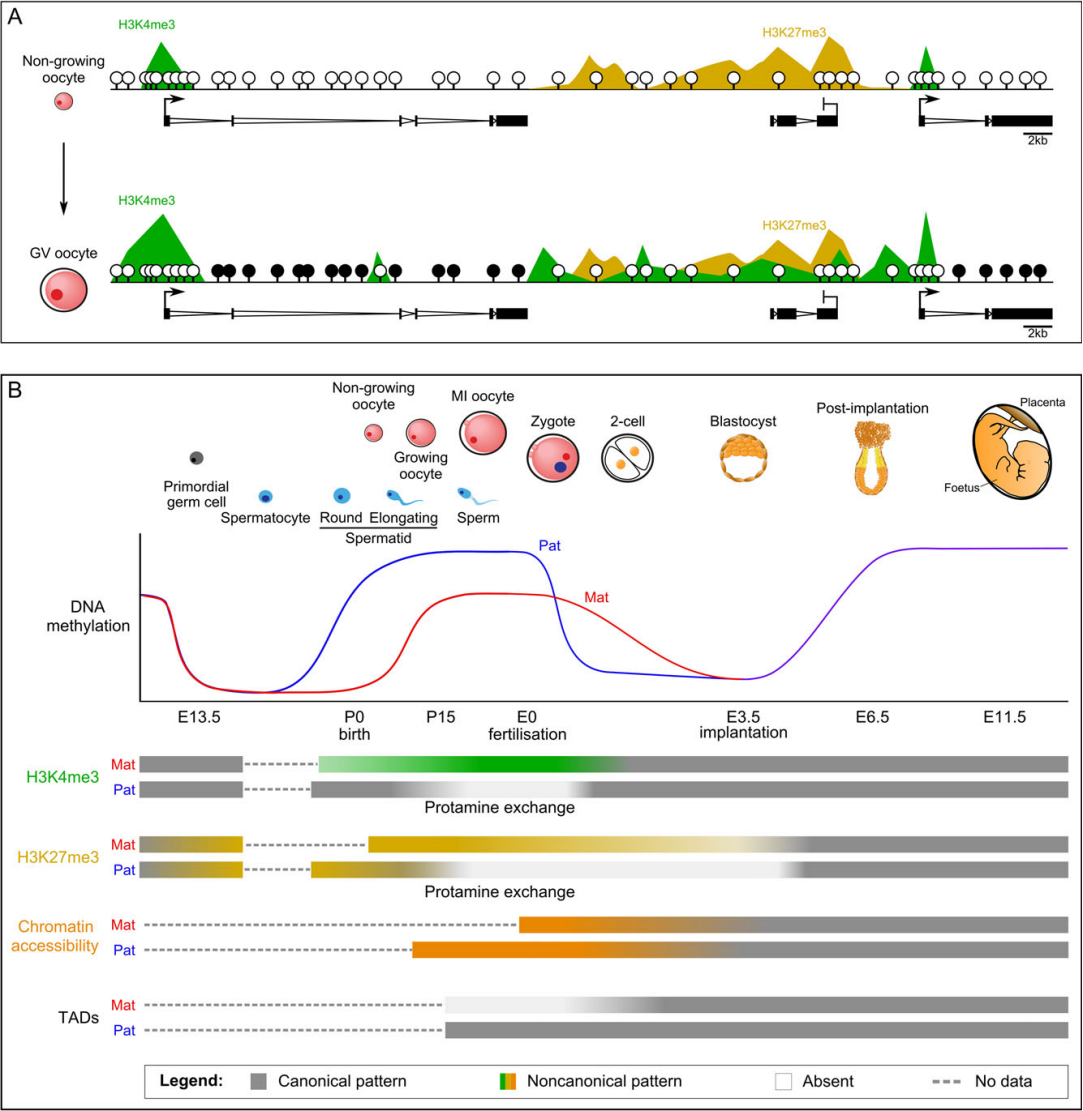
Epigenetic reprogramming in mouse development. (**A**) Epigenetic patterns are shown for non-growing oocytes and fully grown germinal vesicle (GV) oocytes. In non-growing oocytes, DNA is almost completely unmethylated, H3K4me3 is exclusively enriched at active promoters and H3K27me3 is spanning broad non-canonical domains. By the fully grown GV stage, DNA across transcribed gene bodies is fully methylated and H3K4me3 has accumulated in broad domains throughout untranscribed regions. (**B**) Schematic of epigenetic reprogramming events during gametogenesis and embryogenesis. DNA methylation is erased in primordial germ cells and re-established earlier in the sperm of males and after birth in oocytes in females. Oocytes acquire lower overall methylation than sperm, with non-canonical genome-wide distribution. After fertilisation, the paternal DNA is rapidly demethylated, while maternal DNA methylation is passively lost over several cell divisions. DNA methylation is re-acquired in canonical patterns in the post-implantation embryo, concomitant with lineage specification. H3K4me3 is non-canonically distributed in the oocyte, is rapidly erased after fertilisation, and becomes canonically enriched at CpG islands and active promoters. Very few domains retain H3K4me3-marked histones in the protamine exchange in sperm and subsequently through the re-acquisition of histones in the zygote. H3K27me3 acquires a non-canonically broad distribution in PGCs in the absence of other repressive epigenetic marks. This pattern is relatively maintained throughout oogenesis, while very few H3K27me3-marked histones are retained in the sperm protamine exchange. In the pre-implantation embryo, H3K27me3-transmitted from the gametes is progressively lost, with pronounced loss at CpG-rich regions. H3K27me3 is then re-established in a canonical pattern in the post-implantation embryo. Chromatin accessibility is contrastingly and exceptionally open in the oocyte and compact in the sperm. The open chromatin state of maternal DNA is gradually resolved in the pre-implantation embryo, while the compact packaging of paternal DNA is rapidly resolved with incorporation of histones in the zygote. Topological associated domains (TADs) are nearly absent in the mature oocyte and become gradually re-instated in the pre-implantation embryo.

The chromatin organisation and epigenetic profiles of the male and female gametes at the time of fertilisation are profoundly different. Sperm DNA is highly methylated and tightly packaged with protamines, a protein that replaces canonical histones (Wright, 1999); while oocyte DNA is uniquely methylated in an bimodal pattern and is associated with non-canonical distributions of histone modifications (Tomizawa *et al.*, 2012) (Fig. 3). These divergent patterns are established during gametogenesis, which is initiated during embryonic development. The precursors for both male and female germ cells are assigned in the epiblast at embryonic day (E) 7.25 and as these primordial germ cells (PGCs) migrate to the genital ridge (E9.5–E11.5), they undergo almost complete demethylation of the genomic DNA (Guibert *et al.*, 2012; Seisenberger *et al.*, 2012). The loss of DNA methylation is due to downregulation of both *de-novo* DNMTs and the DNMT1-cofactor UHRF1 (Kagiwada *et al.*, 2013). With the decline of DNA methylation, there is a re-organisation of repressive histone marks as well, with widespread loss of H3K9me2 and an increase of H3K27me3 (Seki *et al.*, 2005). PGCs then subsequently progress either into spermatogenesis or oogenesis, depending on the sex of the embryo.

In early sperm progenitors (prospermatogonia), DNA methylation begins to be re-established before birth (E15.5–E18.5) and is completed at the termination of meiotic pachytene after birth (D10–19) (Hajkova *et al.*, 2002) (Fig. 3). DNA methylation is essential for meiotic progression (Bourc’his and Bestor, 2004). Methylation of sperm DNA broadly resembles other cell types in that it is almost uniformly methylated with the exception of regulatory regions. While the DNA is initially wrapped around histones in spermatocytes, the vast majority of histones are replaced, first with non-canonical histone variants and transition proteins, which are subsequently replaced with protamines during maturation, allowing the DNA to be tightly packaged into the compact sperm head (Balhorn *et al.*, 2000; Bao and Bedford, 2016). The functional relevance of the ~1% of histones that are retained in mature sperm is still debated. It seems that at least a subset of these histones reside at CpG-rich promoters with low DNA methylation, although it has been suggested that the vast majority are retained in gene poor regions (Erkek *et al.*, 2013; Carone *et al.*, 2014). Residual histones in sperm support the possibility of intergenerational or possibly transgenerational inheritance of an intrinsic epigenetic memory programme through the male germline. Indeed, loss of H3K4me2 in sperm caused by forced expression of an H3K4-demethylase has been shown to impair the viability of offspring in subsequent generations (Siklenka *et al.*, 2015).

Shortly after the migration of germ cells to the gonad in females, there is massive mitotic expansion of this germ cell pool (E11.5). At E13.5, these oocyte precursors enter meiotic arrest in prophase I and remain quiescent in the developing ovary until after birth when they are assembled into primordial follicles. These cells represent the oocyte pool for the female’s entire lifespan, only a small subset of which will ever become fully mature and ovulate, as the vast majority will undergo apoptosis.

During folliculogenesis, oocytes undergo *de-novo* DNA methylation in the phase of oocyte growth (Hiura *et al.*, 2006), mediated by the *de-novo* DNA methyltransferases DNMT3A and cofactor DNMT3L (Bourc’his *et al.*, 2001; Kaneda *et al.*, 2004) (Fig. 3). Unlike the highly methylated sperm, oocyte methylation is distinctly located over transcribed gene bodies (Kobayashi *et al.*, 2012; Veselovska *et al.*, 2015) in a pattern that is unique among mammalian cell types. The acquisition of DNA methylation across transcribed regions has been suggested to be dependent on the modification of associated histones, including acquisition of H3K36me3 and exclusion of H3K4me3 by H3K4 demethylases KDM1A/B (Stewart *et al.*, 2015; Gahurova *et al.*, 2017). DNMT1 and UHRF1 are required to complete *de-novo* methylation, which is unusual as this protein complex normally functions in the context of maintenance methylation (Shirane *et al.*, 2013; Maenohara *et al.*, 2017). Oocytes also have unusually high levels of methylation of cytosines outside of a CpG context. The functional significance of this ‘non-CpG’ methylation is unclear and may merely reflect the protracted period during which the *de-novo* methyltransferases are active (Tomizawa *et al.*, 2012; Shirane *et al.*, 2013). Curiously, DNA methylation in general has no obvious function in oocytes, as loss of DNA methylation through conditional deletion of *Dnmt3a* or *Dnmt3L* has no effect on oogenesis (Kaneda *et al.*, 2004; Bourc’his *et al.*, 2001).

Intriguingly, across the unmethylated fraction of the oocyte genome, histone modification patterns are also non-canonical in their distribution. A histone mark typically associated with active promoters, H3K4me3, accumulates in a transcription-independent manner at unusually broad, inter-genic domains (Dahl *et al.*, 2016; Zhang *et al.*, 2016; Hanna *et al.*, 2018) (Fig. 3). This non-canonical pattern of H3K4me3 has been attributed to the activity of a single H3K4 methyltransferase, MLL2 and appears to be, at least partially, driven by underlying CpG density (Hanna *et al.*, 2018). Paradoxically, acquisition of non-canonical domains of H3K4me3 appears to be required for genome-wide transcriptional silencing associated with oocyte maturation and resumption of meiosis (Andreu-Vieyra *et al.*, 2010; Dahl *et al.*, 2016; Zhang *et al.*, 2016). Repressive H3K27me3, is also found broadly throughout unmethylated genomic regions and appears to be actively excluded from transcribed regions throughout oogenesis (Zheng *et al.*, 2016) (Fig. 3). The role of H3K27me3 in oogenesis is not clear, but it appears to be required to establish a non-canonical form of imprinting in the early embryo (Inoue *et al.*, 2017a), discussed in more detail below.

The oocyte also has a very distinct chromosome architecture compared to other cell types. Chromatin undergoes major conformational changes during the final stages of maturation in the germinal vesicle (GV) oocyte, from a non-surrounded nucleolar-like body (NSN) to a surrounded (SN) state (Mattson and Albertini, 1990; Zuccotti *et al.*, 1995) accompanying transcriptional silencing. In GV oocytes, Hi-C studies have found chromosome interactions such as TADs and chromosome loops, but the strength of these interactions begin to decrease as the oocytes progress through the NSN to SN transition (Flyamer *et al.*, 2017). With resumption of meiosis, oocytes appear to lose all higher-order chromatin structures, such that metaphase II (MII) oocytes show a uniform interaction pattern along entire chromosomes that appears to be locus independent (Ke *et al.*, 2017; Du *et al.*, 2017).

The distinct epigenetic patterns observed in the oocyte suggest that there may be an uncoupling of some of the conventional mechanisms of gene regulation. This uncoupling might be a requirement to allow the oocyte to maintain necessary gene regulation, while simultaneously establishing an epigenome capable of facilitating the early events of embryogenesis.

### From germ cells to the embryo

In the zygote, the maternal and paternal genomic contributions are reprogrammed distinctively and these dynamics are required for the acquisition of totipotency and zygotic genome activation (ZGA). Immediately after fertilisation, the paternal protamines are replaced by maternal histones accompanied by widespread erasure of almost all paternal DNA methylation. This was proposed to be active demethylation mediated through TET activity (Gu *et al.*, 2011; Inoue and Zhang, 2011), but recent data challenges this finding (Amouroux *et al.*, 2016) and thus the mechanism of this initial erasure remains unresolved (Hill *et al.*, 2014). Conversely, maternal DNA methylation is largely preserved at this stage. However, it does appear that the widespread, non-canonical maternal H3K4me3 needs to be reprogrammed in order for the embryo to initiate ZGA and this occurs through activity of H3K4 demethylases KDM5B and KDM1A (Zhang *et al.*, 2016; Dahl *et al.*, 2016; Ancelin *et al.*, 2016).

Long-range and local chromosome interactions are not immediately restored in the post-meiotic zygote, as is the case for mitotic cells. Intriguingly, re-establishment of higher-order chromatin structure occurs independently of ZGA and cell cycle, suggesting that additional factors are required for re-establishing these interactions (Du *et al.*, 2017). Chromosome compartmentalisation in zygotes is associated with DNA methylation, chromatin accessibility and H3K27me3, but not broad maternal H3K4me3 (Ke *et al.*, 2017).

As the embryo develops towards the blastocyst stage, DNA methylation is passively lost from both the maternal and paternal genomes, resulting in the erasure of most gametic DNA methylation. There are a few thousand domains that are protected from this erasure; these include, but are not limited to, imprinted domains and some classes of repetitive elements (Smallwood *et al.*, 2011). Similar to DNA methylation, repressive H3K27me3 also appears to be progressively lost during pre-implantation development, with maternal H3K27me3 being preferentially retained at distal, inter-genic regions (Zheng *et al.*, 2016). The mechanism for preferential loss or retention of maternal H3K27me3 at specific loci remains unclear. In *Drosophila*, maternally inherited H3K27me3 regulates the activation of enhancers in the early embryo (Zenk *et al.*, 2017). Considering the correlation between compartmentalisation, chromatin accessibility and H3K27me3 (Ke *et al.*, 2017), loss of H3K27me3 may also be required for the establishment of promoter–enhancer interactions in mammalian pre-implantation development. H3K9 di- and tri-methylation are repressive histone modifications that are tightly associated with DNA methylation and are bound by heterochromatin protein 1 (HP1) (Bannister *et al.*, 2001; Lachner *et al.*, 2001); however, there is currently no molecular data evaluating the dynamics of H3K9 methylation in oocytes or early embryogenesis. Immunofluorescence shows that H3K9me3, typically associated with silenced repetitive DNA, is predominantly inherited at maternal peri-centromeres in the early embryo (Puschendorf *et al.*, 2008). As the paternal chromatin structure is newly re-established with the re-integration of histones, the peri-centromeres are first silenced by H3K7me3 and by the 8-cell stage similarly acquire H3K9me3 (Puschendorf *et al.*, 2008). In addition to these repetitive regions, H3K9me2 and H3K9me3 may be required to maintain silencing and protect parental DNA methylation at imprinted domains (Nakamura *et al.*, 2007; Quenneville *et al.*, 2011), as discussed in more detail below. Future characterisation of the genomic distribution of H3K9me2/3 will be essential to determine the role for these marks in early gene regulation and protection of germline DNA methylation.

In addition to DNA methylation and histone remodelling in pre-implantation development, chromatin structure appears to be progressively re-organised. ATAC-seq (Wu *et al.*, 2016) and Hi-C experiments (Du *et al.*, 2017; Flyamer *et al.*, 2017; Ke *et al.*, 2017) showed that zygotes have a very relaxed chromatin state, which is gradually resolved to a more canonical state by the blastocyst stage, a finding that is consistent with previous microscopy-based observations (Ahmed *et al.*, 2010; Burton and Torres-Padilla, 2014). With the re-establishment of higher-order chromatin structure in the pre-implantation embryo, interactions between promoters and enhancers become defined (Du *et al.*, 2017; Ke *et al.*, 2017) and the number of DNase hypersensitivity sites increases (Lu *et al.*, 2016).

Together, the epigenetic profiles explored to date in pre-implantation embryos demonstrate that the chromatin regulatory landscape is dynamic during the transition from a totipotent to a pluripotent embryo with refinement of chromatin compartments and localisation of H3K4me3 to promoters. Paradoxically, this transition is accompanied by almost widespread loss of repressive DNA methylation and H3K27me3, suggesting that targeting of transcriptional machinery in pre-implantation embryo is not facilitated by these protective repressive marks.

### Lineage specification in post-implantation development

Once the embryo implants, there are widespread morphological changes as cell lineages differentiate, accompanied by epigenetic programming. The role of epigenetic regulation during this lineage specification is complex and still not fully understood. Many studies investigating epigenetic mechanisms in lineage specification thus far have used transgenic mouse models to identify key regulators; *in-vivo* data showing the localisation and dynamics of epigenetic modifications remain scarce.

There is substantial evidence for a function for repressive chromatin marks in reinforcing lineage specification. During post-implantation development, there is *de-novo* acquisition of repressive DNA methylation (Okano *et al.*, 1999), H3K9me2 (Zylicz *et al.*, 2015) and H3K27me3 (Zheng *et al.*, 2016), all of which are essential for appropriate lineage development. Genetic ablation in mice of the H3K27 methyltransferase EZH2 (O’Carroll *et al.*, 2001), H3K9 methyltransferase EHMT2 (also known as G9A) (Tachibana *et al.*, 2002, 2005) or *de-novo* DNA methyltransferase DNMT3B (Okano *et al.*, 1999) all lead to developmental abnormalities and lethality in mid-gestation.

In the post-implantation embryo, repressive H3K27me3 is targeted *de novo* to transcriptionally silent promoters, including CpG islands, and gene bodies, by Polycomb repressive complex proteins (Liu *et al.*, 2016; Zheng *et al.*, 2016) and is required to keep these genes transcriptionally repressed at this stage (Yang *et al.*, 2018). This programming corresponds to a widespread switch from the maternally inherited enrichment pattern at silent inter-genic B compartments in the pre-implantation embryo to the regulatory domains of active A compartments in the post-implantation embryo (Zheng *et al.*, 2016; Ke *et al.*, 2017). At many regulatory domains, H3K27me3 can be found together with H3K4me3 in a chromatin state referred to as bivalent. Bivalent domains are enriched at unmethylated, but silent, promoters of developmental genes in the post-implantation epiblast and extra-embryonic ectoderm (Rugg-Gunn *et al.*, 2010). Bivalent chromatin is thought to poise these genes for rapid activation or repression during lineage specification in the developing embryo (Bernstein *et al.*, 2006). Indeed, it has been shown during migration and development of neural crest cells that bivalent genes are embedded in large repressive Polycomb domains in which they maintain plasticity and chromatin accessibility in all subpopulations (Minoux *et al.*, 2017). Upon differentiation, decreasing H3K27me3 and increasing H3K4me2 then leads to cell type-specific gene expression (Minoux *et al.*, 2017). While the genomic location and resolution of bivalent domains has now been characterised *in vivo* (Minoux *et al.*, 2017; Zheng *et al.*, 2016), paralleling the observations made in cultured cells, it remains unclear how H3K27me3 is targeted in the post-implantation embryo.

Unlike the targeted gain of H3K27me3 in the post-implantation embryo, DNA methylation is established across ~80% of the embryonic genome. Yet despite its widespread occurrence, DNA methylation does not appear to be necessary to direct the transcriptional programme in early embryos, but rather to reinforce lineage decisions (Zhang *et al.*, 2018). As such, there are a few key domains that become differentially methylated between the post-implantation embryonic and extra-embryonic compartments to prevent aberrant trans-differentiation (Ng *et al.*, 2008; Zhang *et al.*, 2018). Similarly, differential methylation was observed at functionally relevant enhancer elements between gastrulating tissues, suggesting that this mechanism may also reinforce lineage commitment within the embryo (Zhang *et al.*, 2018).

Interestingly, despite dramatic acquisition of H3K9me2 in post-implantation development, it is not required for the genome-wide gain of DNA methylation, but rather appears to be important for a small subset of CpG-rich domains (Auclair *et al.*, 2016). As such, deposition of H3K9me2 is only necessary for efficient repression of a few germline-specific genes, mediated by silencing of their promoters and/or enhancers (Zylicz *et al.*, 2015; Auclair *et al.*, 2016). Furthermore, H3K9me2 deposited by EHMT2 is not required for silencing the vast majority of repetitive elements (Zylicz *et al.*, 2015). Together these findings suggest that H3K9me2 is ubiquitously associated with methylated DNA in the post-implantation embryo, but its functional role is rather specialised. This may be attributable to redundancies in repressive epigenetic marks or to these repressive modifications acting not as upstream transcriptional regulators but rather as reinforcements for transcriptionally inactive regions of DNA.

Active chromatin marks, such as H3K4me3 and H3K27ac, also likely play a role in transcriptional regulation during lineage specification. Active histone modifications can promote transcription by facilitating the accessibility of regulatory regions to transcription factors, but whether these marks are required for establishing a transcriptional programme or for merely reinforcing it remains contentious (Howe *et al.*, 2017). For example, the level of H3K4me3 at promoters correlates with transcription and transcriptional machinery interacts with H3K4me3; however, in many contexts, ablation of H3K4me3 has a limited effect on transcription (Briggs *et al.*, 2001; Clouaire *et al.*, 2012; Margaritis *et al.*, 2012). The predominant H3K4 methyltransferase in the pre-implantation embryo is MLL2, while in the post-implantation embryo it is SETD1A (Bledau *et al.*, 2014). However, due to the overlapping redundancy of the six H3K4 methyltransferase proteins, it has been challenging to interpret the role for each methyltransferase and, in turn, H3K4me3 during embryogenesis (Bledau *et al.*, 2014). Embryos deficient in H3K4 methyltransferases MLL1 and MLL2 both arrest in mid-gestation and show patterning defects likely due to aberrant expression levels of a subset of the *Hox* genes (Ernst *et al.*, 2004; Glaser *et al.*, 2006), suggesting that MLL1/2-mediated H3K4me3 is required to express appropriate levels of these genes. The post-implantation upregulation of SETD1A appears to have a central role in lineage specification, as it is required to complete gastrulation (Bledau *et al.*, 2014), suggesting that it may be important for establishing transcriptional patterning.

During differentiation, acquisition of H3K27ac at enhancers is associated with the formation of enhancer-promoter interactions and induction of their target genes (Wang *et al.*, 2016; Rubin *et al.*, 2017). Knockout in mice of the H3K27 acetyltransferases CBP or p300 (which also acetylate other histone residues and interact with many transcription factors themselves) leads to mid-gestation embryonic lethality (Yao *et al.*, 1998). Embryos suffer from neural tube defects and aberrant cell proliferation (Yao *et al.*, 1998); surprisingly, this suggests that H3K27ac is not required for establishing the transcriptional programming during early lineage specification. This is consistent with the finding that the effect of histone acetylation on chromatin accessibility is rather subtle (Wang *et al.*, 2000). Therefore, H3K27ac may act synergistically to increase chromatin accessibility at active regulatory elements, but likely is not sufficient to activate a locus.

Together these studies suggest that several epigenetic marks, in particular H3K27me3 and H3K4me3, are required for lineage specification, but for the most part it appears that epigenetic modifications may reinforce lineage commitment rather than direct it. As new single/low-cell molecular approaches are implemented to evaluate gene regulation and epigenetic patterning through this important developmental window, new insights into the molecular hierarchy of gene regulation may be revealed.

### Dynamics of genomic imprinting during embryonic development

As discussed above, a subset of genomic loci maintain gamete DNA methylation throughout epigenetic reprogramming in the embryo. These domains are termed germline differentially methylated regions (gDMRs) and their mono-allelic parent-of-origin DNA methylation persists through cell differentiation and into adulthood. GDMRs can regulate nearby genes, resulting in mono-allelic gene expression, termed genomic imprinting. In mice, there are 23 maternal and three paternal gDMRs regulating the gene expression of ~151 genes (https://www.mousebook.org/imprinting-gene-list). Collectively, imprinted genes are essential for development, as demonstrated by embryo manipulation experiments used to generate embryos with exclusively maternal or paternal genomes (McGrath and Solter, 1984; Surani *et al.*, 1984). These embryos showed severe developmental and placental defects and do not survive.

There has been extensive investigation into the mechanisms allowing gDMRs to evade the DNA methylation erasure in the pre-implantation embryo. Several essential proteins have been identified, including DNA-binding proteins (ZFP57, UHRF1), key interactors (TRIM28/KAP1) and histone binding proteins (PGC7/Stella) (Bostick *et al.*, 2007; Sharif *et al.*, 2007; Li *et al.*, 2008; Quenneville *et al.*, 2011; Messerschmidt *et al.*, 2012; Nakamura *et al.*, 2012). These appear to assemble in a complex that facilitates recruitment of DNMT1 and the H3K9 methyltransferase SETDB1 and exclusion of DNA demethylation enzymes (TETs) at imprinted gDMRs (Messerschmidt, 2012).

A recent study has also shown that genomic imprinting can be conferred by another epigenetic mark in addition to DNA methylation: maternal H3K27me3 inherited from the oocyte (Inoue *et al.*, 2017a). Inoue and colleagues (2017a) identified several domains where the maternal allele was silenced by H3K27me3, thereby mediating paternal-specific gene expression. Intriguingly, this non-canonical form of imprinting was only able to be maintained in extra-embryonic lineages post-implantation (Inoue *et al.*, 2017a), suggesting embryonic lineages effectively reprogram the parental bias at these domains.

## Human development: how conserved are mechanisms between mouse and human?

Using low-input and single-cell sequencing techniques, the first advances with human samples were made in recent years, allowing us to compare the transcriptome, methylome and chromatin accessibility of human gametes and early embryos with what is known from mouse models. Other technologies, such as Hi-C, are likely to follow soon, but current ChIP-seq methods still require at least one hundred cells, making progress with human oocytes and early cleavage stage embryos more challenging. So far, studies on human development have shown that, in general, DNA methylation patterns and reprogramming events are relatively conserved between mouse and human (Table II). This supports the mouse as a model organism for elucidating general mechanisms of epigenetic reprogramming in early development. However, when looking in detail, differences can be observed, likely with functional consequences (Table II). In the following sections, we highlight the known differences between human and mouse.

**Table II:** Comparative evaluation of epigenetic features and processes evaluated during human and mouse development to date.

Tissue/cell type	Epigenetic feature/process	Mouse	Human	Reference	Relative similarity
PGCs	DNA methylation erasure	Global DNA methylation and imprinted DMRs are erased upon PGC specification	Global DNA methylation and imprinted DMRs are erased upon PGC specification	Guibert *et al.* (2012), Seisenberger *et al.* (2012), Guo *et al.* (2015), Gkountela *et al.* (2015), Guo *et al.* (2017b)	
Sperm	DNA methylation patterns in sperm	~80% genome-wide methylation, with unmethylated regulatory domains	~75% genome-wide methylation, with unmethylated regulatory domains	Oakes *et al.* (2007), Kobayashi *et al.* (2012), Guo *et al.* (2014)	
	*De novo* DNMTs in spermatogenesis	DNMT3A, 3L and 3C are essential for spermatogenesis	Unknown; DNMT3A, 3B and 1 are dynamically expressed during spermatogenesis, but there is no expression of DNMT3L and no orthologous gene for DNMT3C	Bourc’his *et al.* (2001), Kaneda *et al.* (2004), Barau *et al.* (2016), Marques *et al.* (2011)	
	Retention of modified histones in sperm	~1% genome-wide, enriched at developmental promoters	~10% genome-wide, enriched at developmental promoters	Brykczynska *et al.* (2010), Hammoud *et al.* (2009)	
Oocyte	DNA methylation patterns in the oocyte	~40% genome-wide methylation and localised predominantly to expressed gene bodies	~54% genome-wide methylation and localised predominantly to gene bodies	Okae *et al.* (2014), Kobayashi *et al.* (2012)	
	*De novo* DNMTs in oogenesis	DNMT3A and 3L are essential for establishing DNA methylation in oocytes	Unknown; in human oocytes, DNMT1, 3A and 3B are expressed, but not DNMT3L	Bourc’his *et al.* (2001), Smallwood *et al.* (2011), Shirane *et al.* (2013), Guo *et al.* (2014), Okae *et al.* (2014)	
	Histone modification patterns	Non-canonical distributions of both H3K4me3 and H3K27me3 across regions lacking DNA methylation	Unknown	Zhang *et al.* (2016), Dahl *et al.* (2016), Hanna *et al.* (2018), Zheng *et al.* (2016)	
	Higher order chromatin organisation	Weak TADs and loops and a loss of A/B compartments upon transcriptional silencing	Unknown	Flyamer *et al.* (2017), Du *et al.* (2017), Ke *et al.* (2017)	
Pre-implantation embryo	DNA methylation dynamics in pre-implantation development	Active loss of paternal methylation and passive loss of maternal methylation; regions of DNA methylation turnover	Active loss of paternal methylation and minimal passive loss of maternal methylation; regions of DNA methylation turnover	Guo *et al.* (2014), Okae *et al.* (2014), Smith *et al.* (2014), Zhu *et al.* (2018)	
	ZFP57-mediated protection of imprinted DMRs	Maternal/oocyte contribution of ZFP57 is required to protect imprints in pre-implantation development	ZFP57 is required to protect imprints, but it is not expressed in human oocytes; expression is initiated in the pre-implantation embryo	Quenneville *et al.* (2011), Li *et al.* (2008), Okae *et al.* (2014), Mackay *et al.* (2008), Sanchez-Delgado *et al.* (2016b)	
	Chromatin configuration post-fertilisation	Widespread open chromatin that resolves upon ZGA	Widespread open chromatin that resolves upon ZGA	Wu *et al.* (2016), Wu *et al.* (2018)	
	Histone modification dynamics	Non-canonical maternal H3K4me3 resolves to canonical pattern, while maternal H3K27me3 is predominantly erased	Unknown	Zheng *et al.* (2016), Zhang *et al.* (2016), Dahl *et al.* (2016)	
	Higher order chromatin organisation	Canonical patterns of TADs, loops, and A/B compartments restored during early embryogenesis	Unknown	Flyamer *et al.* (2017), Ke *et al.* (2017), Du *et al.* (2017)	
Blastocyst	DNA methylation patterns in blastocyst-stage embryos	Maintenance of imprinted DMRs and low levels of oocyte methylation patterns	Maintenance of imprinted DMRs and persistent oocyte methylation patterns	Kobayashi *et al.* (2012), Okae *et al.* (2014), Guo *et al.* (2014), Zhu *et al.* (2018)	
Post-implantation embryonic tissues	Number of imprinted genes	~125–151, with numerous imprinted gene clusters	~50–90, with numerous imprinted gene clusters	Crowley *et al.* (2015), Babak *et al.* (2015), Sanchez-Delgado *et al.* (2016a), Santoni *et al.* (2017), Andergassen *et al.* (2017)	
	Epigenetic regulation of imprinted gene clusters	Non-coding RNAs and differential DNA methylation regulate imprinted gene expression	Non-coding RNAs and differential DNA methylation regulate imprinted gene expression	Reviewed in Reik and Walter (2001)	
	X chromosome inactivation (XCI) in embryogenesis	Random XCI, mediated by opposing expression of *Xist* and *Tsix*	Random XCI, mediated by expression of *XIST* from the inactive X	Reviewed in Furlan and Rougeulle (2016)	
	Genetic polymorphisms influence imprinted gene expression	*Cis*-acting strain-specific SNPs can influence allelic bias in imprinted gene expression	*Cis*-acting SNPs can influence allelic bias in imprinted gene expression	Crowley *et al.* (2015), Andergassen *et al.* (2017), Babak *et al.* (2015), Garg *et al.* (2012)	
	Tissue-specific imprinted gene expression	Several imprinted genes are tissue-specific	Several imprinted genes are tissue-specific	Crowley *et al.* (2015), Andergassen *et al.* (2017), Babak *et al.* (2015)	
Post-implantation extra-embryonic tissues	Genome-wide methylation patterns	Extra-embryonic tissues are characterised by large partially methylated domains	Extra-embryonic tissues are characterised by large partially methylated domains	Rossant *et al.* (1986), Schroeder *et al.* (2013), Decato *et al.* (2017)	
	XCI in extra-embryonic tissues	Imprinted inactivation of the paternal X chromosome, conferred by repression of maternal *Xist* by oocyte-derived H3K27me3	Random XCI	Takagi and Sasaki (1975), Migeon and Do (1979), Penaherrera *et al.* (2003), Inoue *et al.* (2017b)	
	Abundance of placental-specific imprinted gDMRs	None reported	>1500 placental-specific gDMRs reported	Hanna *et al.* (2016), Hamada *et al.* (2016), Sanchez-Delgado *et al.* (2016a)	
	Polymorphic imprinted DMRs	Unknown	Pervasive in extra-embryonic tissues	Hanna *et al.* (2016), Sanchez-Delgado *et al.* (2016a)	
	Non-canonical imprinting	Several non-canonical placenta-specific imprinted genes mediated by maternal H3K27me3	Unknown	Inoue *et al.* (2017a)	
	Large placenta-specific imprinted domains: KvDMR	Distal placental-specific imprinting of genes in the KvDMR locus	While the canonical imprinting at KvDMR is conserved, distal genes are not imprinted in placenta	Lewis *et al.* (2004); Frost *et al.* (2010)	
	Large placenta-specific imprinted domains: Chromsome 19 micro-RNA cluster	No orthologous region	Chromosome 19 micro-RNA cluster is imprinted specifically in placenta	Noguer-Dance *et al.* (2010)	

**Colour key: green** – highly similar; **yellow** – similar, but with key differences identified; **red** – highly discrepant; **grey** – unknown in mouse or human.

### Gametes

Similar to mice, human PGCs undergo almost complete erasure of DNA methylation during early embryonic development (Guo *et al.*, 2015, 2017b; Gkountela *et al.*, 2015). Given the difficulties in obtaining samples from late gestation foetal gonads and immature gametes, the resetting of DNA methylation during spermatogenesis and oogenesis remains unexplored. However, the DNA methylome of human mature gametes, gives us some insights into epigenetic programming events during gametogenesis.

There are substantial physiological differences between mammalian species during spermatogenesis (Ehmcke *et al.*, 2006), and yet, global epigenetic trends in mature sperm, such as DNA hyper-methylation in inter-genic regions and the histone-to-protamine exchange, are similar (Molaro *et al.*, 2011). However, some aspects of the *de-novo* DNA methylation mechanisms may differ between mouse and human. Recently, a novel DNA methyltransferase (DNMT3C) was discovered, specifically active at young transposable elements during mouse spermatogenesis (Barau *et al.*, 2016). In male mice, this enzyme is crucial for fertility, but this gene is not present in the human genome. Furthermore, while DNMT3L is essential for spermatogenesis in mice (Bourc’his *et al.*, 2001), DNMT3L appears to not be expressed at any time during human spermatogenesis (Marques *et al.*, 2011). While the replacement of histones by protamines is conserved, ~10-fold more nucleosomes appear to be retained in human sperm than in mouse sperm (Hammoud *et al.*, 2009; Brykczynska *et al.*, 2010). Retained histones may therefore be more likely to permit paternal epigenetic regulation of transcription in the pre-implantation embryo (Carrell, 2012; Miller *et al.*, 2010), although this remains to be shown.

**Figure 4:**
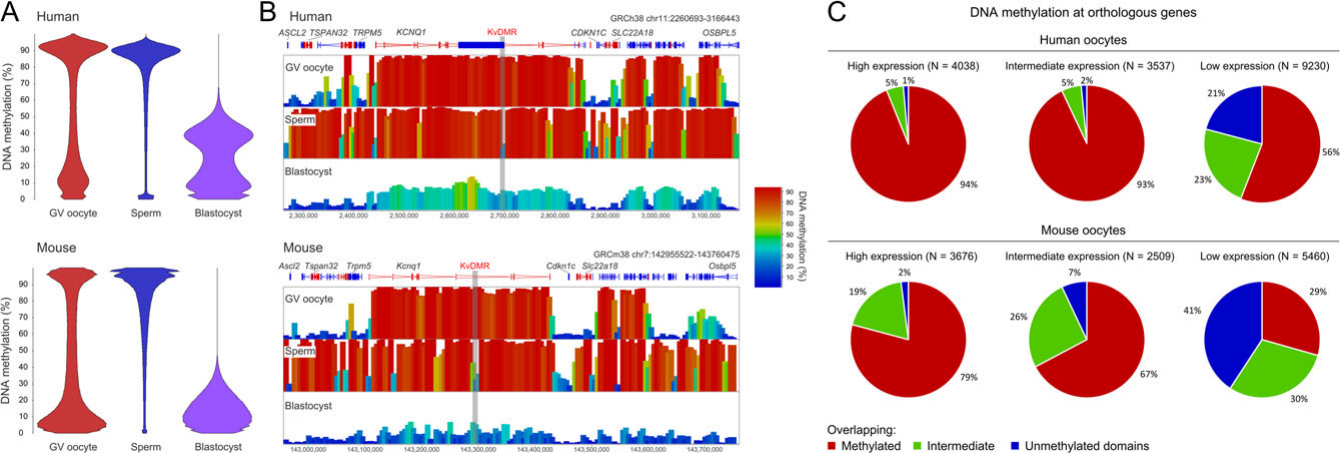
Comparison of DNA methylation in human and mouse development. (**A**) Beanplots showing the distribution of DNA methylation percentages of 100-CpG running windows (minimum coverage of 10 CpGs) in human (top) and mouse (bottom) GV oocytes, sperm and blastocysts, with human oocytes and blastocysts being notably more methylated than mouse oocytes and blastocysts, respectively. (**B**) Screenshot of DNA methylation at the *KvDMR* imprinted locus in human (top) and mouse (bottom) GV oocytes, sperm and blastocysts. The locus illustrates the increased number of regions that are fully methylated in human compared to mouse oocytes. Additionally, the human blastocyst retains the maternal pattern of methylation more substantially than the mouse blastocyst. (**C**) Proportion of orthologous genes that are methylated in human and mouse oocytes. Orthologous genes were defined by ENSEMBL BioMart and categorised as highly expressed (FPKM>10), intermediately expressed (1<FPKM<10) or lowly expressed (FPKM<1). These genes were then evaluated for overlap with fully methylated (>75%) and intermediately methylated (25–75%) 100-CpG windows; genes that did not overlap a methylated window were defined as unmethylated. This analysis demonstrates that the increase in methylated domains in human oocytes reflects an increased number of genes becoming fully methylated compared to mouse. Publically available data was used for these analyses, including RNA-seq data for mouse and human oocytes (GSE44183) (Xue *et al.*, 2013) and DNA methylation data from mouse (Kobayashi *et al.*, 2012) and human (Okae *et al.*, 2014) oocytes, sperm and blastocyst embryos.

The first study to report DNA methylation patterns in human oocytes used RRBS, which mainly captures CpG islands and other CG-rich sequence and covers 5–10% of the genome (Guo *et al.*, 2014). Since then, a genome-wide approach on pools of oocytes and two single-cell studies have been published, all together giving us a very comprehensive understanding of the human fully grown oocyte methylome (Okae *et al.*, 2014; Yu *et al.*, 2017; Zhu *et al.*, 2018). Human oocytes have a higher average DNA methylation level than mice (~54% in humans versus ~40% in mice) (Data source: PRJDB18 and PRJDB4030) (Kobayashi *et al.*, 2012; Okae *et al.*, 2014) (Fig. 4A). Despite the increase in fully methylated regions in human oocytes, it is still predominantly restricted to gene bodies (Fig. 4B). Indeed, a larger proportion of genes are methylated in human oocytes than in mouse (Fig. 4C); however, this is likely not due to an overall increase in transcription in human oocytes, as a similar number of transcripts were detected (FPKM>1) (Data source: GSE44183) (Xue *et al.*, 2013). These findings suggest either that DNMTs may be more active in human oocytes or that the relatively longer duration of oocyte maturation in humans compared to mouse (~150 days vs. 21 days, respectively) permits more extensive accumulation of DNA methylation (Gougeon, 1986; Hiura *et al.*, 2006). Notably, DNMT3L, a co-factor of DNMT3A that is essential for *de-novo* methylation in mouse oocytes (Bourc’his *et al.*, 2001; Smallwood *et al.*, 2011; Shirane *et al.*, 2013), is not expressed in human oocytes (Guo *et al.*, 2014; Okae *et al.*, 2014). It is currently unknown if DNMT3A can function independently in the human oocyte or if it is supported by other factors, like DNMT3B.

### From germ cells to the embryo

After fertilisation, there is global reprogramming of DNA methylation in the human pre-implantation embryo with lowest levels attained at the blastocyst stage (Guo *et al.*, 2014; Okae *et al.*, 2014; Smith *et al.*, 2014; Zhu *et al.*, 2018). The paternal genome is actively demethylated first and more substantially, whereas the maternal genome shows maintenance of much of the oocyte-derived methylation (Guo *et al.*, 2014; Okae *et al.*, 2014; Zhu *et al.*, 2018). The retention of maternal methylation is considerably more substantial in the human than in the mouse, suggesting there is less passive demethylation and that perhaps DNMT1 has a more active role in the human pre-implantation embryo (Fig. 4A and B). As discussed in the section above, in mice, gDMRs are protected from passive demethylation by a complex including ZFP57 (Messerschmidt, 2012). Interestingly, unlike mice, ZFP57 is not expressed in human oocytes (Okae *et al.*, 2014), but is still required for maintenance of imprinting during human development (Mackay *et al.*, 2008). Thus ZFP57 is required for maintaining imprinted gDMRs in both mouse and human, but the developmental stage for its requirement differs.

A profound difference between human and mouse during early pre-implantation development is the discrepancy in timing of ZGA. Whereas the mouse genome undergoes the major wave of ZGA at the 2-cell stage, in human embryos this occurs at the 8-cell stage (Braude *et al.*, 1988; Aoki *et al.*, 1997). Despite these differences in timing, a recent study has shown that pre-ZGA embryos have widespread open chromatin in both mouse and human, and this unusual chromatin landscape is rapidly remodelled upon ZGA (Wu *et al.*, 2018). Importantly, using an inhibitor of transcription (α-amanitin) in mouse and human embryos, they were able to show that this transition of chromatin accessibility was in fact dependent on transcriptional activation (Wu *et al.*, 2018). The widespread open chromatin pattern in transcriptionally silent mouse zygotes has been shown to be linked to non-canonical patterning of H3K4me3 in the oocyte (Dahl *et al.*, 2016; Ancelin *et al.*, 2016; Zhang *et al.*, 2016), and while ChIP-seq data is currently unavailable for human embryos, one can speculate that similar mechanisms may be involved.

In pre-implantation development, there are notable differences in the transcriptome of mouse and human embryos (Xue *et al.*, 2013; Yan *et al.*, 2013; Blakeley *et al.*, 2015). Although similar transcription factors appear to function in mouse and human pre-implantation embryos, the temporal regulation and the transcriptional networks they regulate can differ, suggesting there are divergent aspects of early development (Blakeley *et al.*, 2015; Fogarty *et al.*, 2017; Wu *et al.*, 2018). It is not clear yet if the epigenome may be instructive for some of these transcriptional differences. However, one study found that there was an increasing correlation between transcription and promoter methylation from the zygotic stage to post-implantation, especially after ZGA, in the human embryo (Guo *et al.*, 2014). This suggests that the retention of maternal DNA methylation in the human embryo may play a role.

Initially, pre-implantation development was thought to be exclusively a time of DNA methylation erasure (Smith *et al.*, 2012); however, recent studies in mouse and human show that there is *de-novo* methylation during pre-implantation development (Amouroux *et al.*, 2016; Zhu *et al.*, 2018). Thus far, this phenomenon has been best described in human development. Zhu and colleagues found two phases of *de-novo* methylation: first, the paternal genome in the zygote between the early- to mid-pronuclear stage, just after a major wave of active demethylation; second, between the 4- and 8-cell stage coinciding with ZGA. Regions gaining methylation are enriched in repetitive elements, especially evolutionary younger classes of SINEs and LINEs. The targeting of *de-novo* methylation to potentially more active repeat elements has been suggested to repress their transcriptional activity to avoid mobilisation and safeguard genome stability during ZGA (Smith *et al.*, 2014). However, methylation of these regions was surprisingly transient, as they became demethylated again in the following developmental stages (Zhu *et al.*, 2018). These findings highlight the unexpectedly complex methylation dynamics in the early embryo.

### Post-implantation development

The epigenetic regulation of lineage specification in the post-implantation embryo is largely unexplored in humans due to challenges in obtaining samples. Our current knowledge of epigenetics in post-implantation development is largely extrapolated from human embryonic stem cell differentiation systems, which have provided important insights into tissue differentiation, as discussed elsewhere (Xie *et al.*, 2013). However, recent advances of *in-vitro* culture of human embryos have enlivened the ethical discussion about embryo culture past the implantation-stage blastocyst (Weimar *et al.*, 2013; Shahbazi *et al.*, 2016), and may eventually allow the study of epigenetics in post-implantation development *in vivo*.

### Genomic imprinting

The majority of imprinted gene clusters identified in mouse are conserved in their methylation status, allelic expression and synteny in humans, although with several notable exceptions (Reik and Walter, 2001). There has been considerable work identifying novel imprinted genes in humans, using a combination of sequencing approaches over single nucleotide polymorphisms (SNPs) (Metsalu *et al.*, 2014; Babak *et al.*, 2015; Hamada *et al.*, 2016; Santoni *et al.*, 2017) or cases with genomic imbalances (Choufani *et al.*, 2011; Yuen *et al.*, 2011; Kobayashi *et al.*, 2012; Court *et al.*, 2014; Hanna *et al.*, 2016; Sanchez-Delgado *et al.*, 2016). These studies have estimated there are 50–90 imprinted genes in humans; however, many more DMRs have been identified, but whether these are all regulating gene expression remains unclear. The task of identifying an exhaustive list of imprinted loci in healthy tissues has proven challenging, due to the limited frequency and availability of parental information for SNPs in human populations (Metsalu *et al.*, 2014; Hamada *et al.*, 2016; Santoni *et al.*, 2017), and the pervasive tissue-specific and polymorphic imprinting (Babak *et al.*, 2015; Hanna *et al.*, 2016). Overall, findings suggest that imprinted gene expression and methylation may be more widespread and variable in humans than in mice; however, as similar screens are now being implemented in mice (Babak *et al.*, 2015; Crowley *et al.*, 2015; Andergassen *et al.*, 2017), comparable patterns may emerge.

As discussed in section above, the genome-wide methylation profiles in human and mouse gametes are remarkably similar. This is notable considering that the repertoire of DNMTs responsible for these patterns are not identical. In the foetus and adult, human imprinted genes are similarly regulated by gDMRs that are maintained through early development, yet to date very little is known about how imprints are protected during human reprogramming. Foremost, the maintenance of imprinted gDMRs in the human pre-ZGA embryo appears to be independent of ZFP57 (Okae *et al.*, 2014). However, mutations in *ZFP57* cause 50% of cases with transient neonatal diabetes with loss of imprinting at multiple loci, termed a multilocus imprinting disorder (MLID) (Sanchez-Delgado *et al.*, 2016b), supporting that ZFP57 is required during later stages. Several research groups have sought to identify genetic mutations associated with MLID to identify novel regulators in imprinting in humans; surprisingly, very few genes have been identified (Sanchez-Delgado *et al.*, 2016b). In addition to *ZFP57*, maternal effect genes *NLRP5*, *KHDC3L* and primate-specific *NLRP7* are associated with loss of imprinting (Murdoch *et al.*, 2006; Parry *et al.*, 2011; Docherty *et al.*, 2015). However, these encode cytoplasmic proteins and are thought to be components of the subcortical maternal complex. Therefore, they may be involved in controlling the intracellular localisation of epigenetic regulators in the oocyte or zygote, rather than having a direct role in imprinting (Monk *et al.*, 2017). Together, these findings suggest that the protection of DNA methylation at imprinted gDMRs is required in both mouse and human, but at least some of the epigenetic modifiers may have evolved distinct roles between species.

Recent studies have shown that imprinted gDMRs are far more pervasive in the human placenta than in foetal and adult tissues (Hamada *et al.*, 2016; Hanna *et al.*, 2016; Sanchez-Delgado *et al.*, 2016a). The number of placental-specific imprinted gDMRs is reported to be upwards of 1500, and intriguingly all of these appear to inherit methylation from the oocyte (Hamada *et al.*, 2016). The role of these placental-specific DMRs is still under debate, as many are not associated with genes expressed in the placenta. These domains may therefore be recently evolved imprinted sites (Hanna and Kelsey, 2014), as the vast majority are not conserved between mouse and human (Smith *et al.*, 2014; Hanna *et al.*, 2016). In mouse, placental-specific imprinting appears to be largely conferred by non-canonical repression by maternal H3K27me3 (Lewis *et al.*, 2006; Inoue *et al.*, 2017a), and yet, to date, it is unknown whether humans have this form of non-canonical imprinting.

## Wider implications for human disease and fertility

Recent advances have allowed us to gain the first insights into epigenetic regulation of development, which will be essential in furthering our understanding of the role of epigenetics in human infertility, maternal and foetal health, and complications of pregnancy. As this field develops, it will also become clear whether epigenetic patterns established during prenatal development may influence the lifelong health of offspring and, additionally, whether early epigenetic reprogramming events are susceptible to perturbation by environmental exposures (toxins), physiological factors (stress, diet), or medical interventions (assisted reproductive technologies, ART). In this section, we will provide an overview of the recent developments and future directions in these areas of research.

### Infertility

Evidence from association studies support that aberrant epigenetic programming in sperm may contribute to male infertility. Several studies have found an association between increased histone retention and low sperm count or infertility (Aoki *et al.*, 2005; Torregrosa *et al.*, 2006; Garcia-Peiro *et al.*, 2011; Hammoud *et al.*, 2011; Denomme *et al.*, 2017). Additionally, aberrant sperm DNA methylation patterns has also been associated with semen parameters and male infertility (Montjean *et al.*, 2015; Urdinguio *et al.*, 2015). Furthermore, sequence variants in *DNMT3B* and *DNMT1* have been associated with male infertility (Tang *et al.*, 2017) and variants in *DNMT3L* have been associated with abnormal sperm methylation (Kobayashi *et al.*, 2009).

The role of epigenetics in female infertility has not been evaluated directly due to the invasive procedures required for obtaining oocytes from women. However, there are examples of mutations or genetic anomalies that demonstrate the necessity of oocyte methylation in obtaining a healthy pregnancy (Tomizawa and Sasaki, 2012). Women homozygous for mutations in *NLRP7*, *NLRP5* or *KHDC3L* have pregnancies with a loss of all or some maternal imprints, resulting in recurrent biparental hydatidiform molar pregnancies that miscarry early in development (Murdoch *et al.*, 2006; Parry *et al.*, 2011; Docherty *et al.*, 2015). Furthermore, cases of complete hydatidiform molar pregnancies, in which there is only a paternal genetic contribution, result in no embryo and abnormal placental development (Kajii and Ohama, 1977). Finally, unexplained miscarriage has been associated with defects in imprinted DNA methylation in foetal or placental samples (Hanna *et al.*, 2013; Pliushch *et al.*, 2010; Zheng *et al.*, 2013), which may be a failure to establish imprints or to maintain them. Together, these findings support that gametic epigenetic defects contribute to human infertility and early pregnancy loss; however, the extent to which these changes may be causal in unexplained infertility or subfertility remains unclear.

### Pregnancy complications

Imprinting syndromes are extensively studied developmental epigenetic disorders (Peters, 2014). Loss of allele-specific gene expression at specific imprinted loci can result in developmental defects of varying severity, often involving aberrant foetal growth (reviewed elsewhere; Ishida and Moore, 2013). The role of imprinted genes in foetal growth and placentation (Bartolomei and Ferguson-Smith, 2011) has led to the suggestion that more subtle deregulation of imprinting may contribute to pregnancy complications, such as pre-eclampsia, and/or foetal growth restriction (Frost and Moore, 2010; Moore *et al.*, 2015). However, despite extensive study, many associations remain inconclusive (Koukoura *et al.*, 2012).

The establishment or modulation of post-implantation tissue-specific epigenetic patterns, in particular DNA methylation, have also been widely investigated for association with pregnancy complications. Studies have focused on placental biopsies because of the non-invasive means of obtaining these samples from healthy and pathological pregnancies, as well as the biological relevance (Januar *et al.*, 2015; Robinson and Price, 2015). While many DNA methylation changes have been identified, studies have often been performed on whole placental villi, which can obscure the interpretation of these changes due to cell heterogeneity that may exist between patient groups (Januar *et al.*, 2015). Additionally, DNA methylation changes may be a cause or a consequence of poor placental and/or foetal development. Therefore, an optimised study design will be required to determine whether epigenetic variation can predispose to adverse pregnancy outcomes and, furthermore, whether these changes mediate environmental influences.

### Environmental and physiological influences on epigenetic reprogramming events

The Developmental Origins of Health and Disease (DoHaD) hypothesis posits that adaptive and maladaptive changes during foetal development in response to environmental exposures can result in predisposition to disease in adulthood (Wadhwa *et al.*, 2009). A well-known example of this is the severe prenatal caloric restriction that took place during the Dutch famine, which resulted in increased risk for obesity and comorbidities late in life (Roseboom *et al.*, 2006). It has been suggested that DoHaD effects could be mediated by epigenetic programming in response to these environmental cues.

Investigations into the effects of maternal diet, smoking and stress on DNA methylation in offspring support this idea (Joubert *et al.*, 2012; Dominguez-Salas *et al.*, 2014; Novakovic *et al.*, 2014; Kupers *et al.*, 2015; Palma-Gudiel *et al.*, 2015; Geraghty *et al.*, 2016), while other investigations, such as studies of maternal alcohol consumption, have found no association (Sharp *et al.*, 2018). A particularly compelling example, is the evaluation of maternal diet on foetal DNA methylation in Gambian rural communities. These populations experience profound seasonal fluctuations in nutrient and micro-nutrient availability, and it was found that maternal nutrient status was predictive of DNA methylation patterns of so-called metastable epialleles (genomic loci whose methylation varies between individuals in the absence of genetic variants), including the imprinted gene *VTRNA2-1* (Dominguez-Salas *et al.*, 2014). A challenge for many studies is the interpretation of observed methylation changes, as they are often subtle differences and at only a few CpG sites. Therefore, complementary studies in mouse models are essential to evaluate whether DNA methylation changes due to *in-utero* environmental exposures can influence gene expression patterns and developmental progression (Waterland and Jirtle, 2003).

Additional evidence that early epigenetic programming is susceptible to environmental factors comes from the study of pregnancy outcomes associated with ART. With the increase in ART use globally, there has been an extensive effort to evaluate whether procedures, such as ovarian stimulation, *in-vitro* fertilisation (IVF), intra-cytoplasmic sperm injection (ICSI) and *in-vitro* culture, may increase the risk of developmental epigenetic defects (Canovas *et al.*, 2017). ART procedures have been reproducibly associated with increased risk of imprinting syndromes in human epidemiology studies, although the prevalence is still extremely low (Hiura *et al.*, 2014; White *et al.*, 2015). Studies directly evaluating epigenetic patterns in ART-generated human embryos are scarce and since *in-vivo* samples as a comparison group are inaccessible, results can be difficult to interpret. Nevertheless, targeted assessment of imprinted gDMRs in human cultured embryos has shown that aberrant imprinting could be present in >50% (White *et al.*, 2015). It remains contentious whether ART is associated with additional pregnancy complications or long-term consequences for offspring health (Davies *et al.*, 2012; Hart and Norman, 2013; Liu *et al.*, 2015; Fauser *et al.*, 2014), and whether these may be due to developmental epigenetic changes remains to be explored.

## Concluding remarks

The recent advances in low-input technologies has provided novel insights into epigenetic dynamics during oogenesis and the earliest events of embryonic development in mice. Studies to date have revealed the unique epigenetic landscape of the oocyte, not only DNA methylation, but also histone modifications and nuclear organisation. Future work will continue to explore the underlying mechanisms and the functional importance of these non-canonical patterns. The evaluation of epigenetic profiles in the early embryo suggest that there is widespread erasure of gametic epigenetic patterns after fertilisation and subsequent re-establishment of DNA methylation, histone modifications, chromatin accessibility and nuclear organisation. While the mechanisms driving this reprogramming are unclear, it is apparent that there are localised exceptions, including both canonical and non-canonical imprinted regions.

At present, the study of epigenetic reprogramming events in humans has been largely restricted to DNA methylation. In comparison to mice, there appear to be similar dynamics in both gametes and the early embryo, and yet the proteins modulating these dynamics are often divergent in timing or function. Thus, future investigations of epigenetic patterns in human development may not only reveal further novel regulatory mechanisms, but also differences in the extent of epigenetic information transmitted from gametes to embryos. These discoveries will be essential in understanding the influence of our environment on pregnancy and lifelong health of offspring.

## Authors’ roles

C.H. and H.D. reviewed the literature, wrote the manuscript sections and generated the figures. G.K. provided input into manuscript content and composition, revised the manuscript and financially supported this work.

## Funding

Grants from the UK Medical Research Council and Biotechnology and Biological Sciences Research Council awarded to G.K. supported this work.

## Conflict of interest

The authors have no conflicts of interest.

## References

[dmy021C1] Ahmed K, Dehghani H, Rugg-Gunn P, Fussner E, Rossant J, Bazett-Jones DP Global chromatin architecture reflects pluripotency and lineage commitment in the early mouse embryo. PLoS One 2010;5:e10531. 20479880 10.1371/journal.pone.0010531 PMC2866533

[dmy021C2] Allis CD, Jenuwein T The molecular hallmarks of epigenetic control. Nat Rev Genet 2016;8:487–500. 10.1038/nrg.2016.59 27346641

[dmy021C3] Amouroux R, Nashun B, Shirane K, Nakagawa S, Hill PW, D’Souza Z, Nakayama M, Matsuda M, Turp A, Ndjetehe E et al De novo DNA methylation drives 5hmC accumulation in mouse zygotes. Nat Cell Biol 2016;2:225–233. 10.1038/ncb3296 PMC4765106 26751286

[dmy021C4] Ancelin K, Syx L, Borensztein M, Ranisavljevic N, Vassilev I, Briseno-Roa L, Liu T, Metzger E, Servant N, Barillot E et al Maternal LSD1/KDM1A is an essential regulator of chromatin and transcription landscapes during zygotic genome activation. Elife 2016;5:e08851. 26836306 10.7554/eLife.08851 PMC4829419

[dmy021C5] Andergassen D, Dotter CP, Wenzel D, Sigl V, Bammer PC, Muckenhuber M, Mayer D, Kulinski TM, Theussl HC, Penninger JM et al Mapping the mouse Allelome reveals tissue-specific regulation of allelic expression. Elife 2017;6:e25125. 28806168 10.7554/eLife.25125 PMC5555720

[dmy021C6] Andreu-Vieyra CV, Chen R, Agno JE, Glaser S, Anastassiadis K, Stewart AF, Matzuk MM MLL2 is required in oocytes for bulk histone 3 lysine 4 trimethylation and transcriptional silencing. PLoS Biol 2010;8:e1000453. 20808952 10.1371/journal.pbio.1000453 PMC2923083

[dmy021C7] Aoki VW, Moskovtsev SI, Willis J, Liu L, Mullen JB, Carrell DT DNA integrity is compromised in protamine-deficient human sperm. J Androl 2005;6:741–748. 10.2164/jandrol.05063 16291969

[dmy021C8] Aoki F, Worrad DM, Schultz RM Regulation of transcriptional activity during the first and second cell cycles in the preimplantation mouse embryo. Dev Biol 1997;2:296–307. 10.1006/dbio.1996.8466 9013938

[dmy021C9] Auclair G, Borgel J, Sanz LA, Vallet J, Guibert S, Dumas M, Cavelier P, Girardot M, Forne T, Feil R et al EHMT2 directs DNA methylation for efficient gene silencing in mouse embryos. Genome Res 2016;2:192–202. 10.1101/gr.198291.115 PMC4728372 26576615

[dmy021C10] Babak T, DeVeale B, Tsang EK, Zhou Y, Li X, Smith KS, Kukurba KR, Zhang R, Li JB, van der Kooy D et al Genetic conflict reflected in tissue-specific maps of genomic imprinting in human and mouse. Nat Genet 2015;47:544–549. 25848752 10.1038/ng.3274 PMC4414907

[dmy021C11] Balhorn R, Brewer L, Corzett M DNA condensation by protamine and arginine-rich peptides: analysis of toroid stability using single DNA molecules. Mol Reprod Dev 2000;2:230–234. 10.1002/(SICI)1098-2795(200006)56:2+<230::AID-MRD3>3.0.CO;2-V 10824973

[dmy021C12] Bannister AJ, Zegerman P, Partridge JF, Miska EA, Thomas JO, Allshire RC, Kouzarides T Selective recognition of methylated lysine 9 on histone H3 by the HP1 chromo domain. Nature 2001;6824:120–124. 10.1038/35065138 11242054

[dmy021C13] Bao J, Bedford MT Epigenetic regulation of the histone-to-protamine transition during spermiogenesis. Reproduction 2016;5:R55–R70. 10.1530/REP-15-0562 PMC4896072 26850883

[dmy021C14] Barau J, Teissandier A, Zamudio N, Roy S, Nalesso V, Herault Y, Guillou F, Bourc’his D The DNA methyltransferase DNMT3C protects male germ cells from transposon activity. Science 2016;6314:909–912. 10.1126/science.aah5143 27856912

[dmy021C15] Barski A, Cuddapah S, Cui K, Roh TY, Schones DE, Wang Z, Wei G, Chepelev I, Zhao K High-resolution profiling of histone methylations in the human genome. Cell 2007;4:823–837. 10.1016/j.cell.2007.05.009 17512414

[dmy021C16] Bartolomei MS, Ferguson-Smith AC Mammalian genomic imprinting. Cold Spring Harb Perspect Biol 2011;7:a002592. 10.1101/cshperspect.a002592 PMC3119911 21576252

[dmy021C17] Baubec T, Colombo DF, Wirbelauer C, Schmidt J, Burger L, Krebs AR, Akalin A, Schubeler D Genomic profiling of DNA methyltransferases reveals a role for DNMT3B in genic methylation. Nature 2015;7546:243–247. 10.1038/nature14176 25607372

[dmy021C18] Bernstein BE, Mikkelsen TS, Xie X, Kamal M, Huebert DJ, Cuff J, Fry B, Meissner A, Wernig M, Plath K et al A bivalent chromatin structure marks key developmental genes in embryonic stem cells. Cell 2006;2:315–326. 10.1016/j.cell.2006.02.041 16630819

[dmy021C19] Blakeley P, Fogarty NM, del Valle I, Wamaitha SE, Hu TX, Elder K, Snell P, Christie L, Robson P, Niakan KK Defining the three cell lineages of the human blastocyst by single-cell RNA-seq. Development 2015;18:3151–3165. 10.1242/dev.123547 PMC4582176 26293300

[dmy021C20] Bledau AS, Schmidt K, Neumann K, Hill U, Ciotta G, Gupta A, Torres DC, Fu J, Kranz A, Stewart AF et al The H3K4 methyltransferase Setd1a is first required at the epiblast stage, whereas Setd1b becomes essential after gastrulation. Development 2014;5:1022–1035. 10.1242/dev.098152 24550110

[dmy021C21] Boers R, Boers J, de Hoon B, Kockx C, Ozgur Z, Molijn A, van IJcken W, Laven J, Gribnau J Genome-wide DNA methylation profiling using the methylation-dependent restriction enzyme LpnPI. Genome Res 2018;1:88–99. 10.1101/gr.222885.117 PMC5749185 29222086

[dmy021C22] Bostick M, Kim JK, Esteve PO, Clark A, Pradhan S, Jacobsen SE UHRF1 plays a role in maintaining DNA methylation in mammalian cells. Science 2007;5845:1760–1764. 10.1126/science.1147939 17673620

[dmy021C23] Bourc’his D, Bestor TH Meiotic catastrophe and retrotransposon reactivation in male germ cells lacking Dnmt3L. Nature 2004;7004:96–99. 10.1038/nature02886 15318244

[dmy021C24] Bourc’his D, Xu GL, Lin CS, Bollman B, Bestor TH Dnmt3L and the establishment of maternal genomic imprints. Science 2001;5551:2536–2539. 10.1126/science.1065848 11719692

[dmy021C25] Boyle AP, Davis S, Shulha HP, Meltzer P, Margulies EH, Weng Z, Furey TS, Crawford GE High-resolution mapping and characterization of open chromatin across the genome. Cell 2008;2:311–322. 10.1016/j.cell.2007.12.014 PMC2669738 18243105

[dmy021C26] Braude P, Bolton V, Moore S Human gene expression first occurs between the four- and eight-cell stages of preimplantation development. Nature 1988;6163:459–461. 10.1038/332459a0 3352746

[dmy021C27] Briggs SD, Bryk M, Strahl BD, Cheung WL, Davie JK, Dent SY, Winston F, Allis CD Histone H3 lysine 4 methylation is mediated by Set1 and required for cell growth and rDNA silencing in *Saccharomyces cerevisiae*. Genes Dev 2001;24:3286–3295. 10.1101/gad.940201 PMC312847 11751634

[dmy021C28] Brind’Amour J, Liu S, Hudson M, Chen C, Karimi MM, Lorincz MC An ultra-low-input native ChIP-seq protocol for genome-wide profiling of rare cell populations. Nat Commun 2015;6:6033. 25607992 10.1038/ncomms7033

[dmy021C29] Brykczynska U, Hisano M, Erkek S, Ramos L, Oakeley EJ, Roloff TC, Beisel C, Schubeler D, Stadler MB, Peters AH Repressive and active histone methylation mark distinct promoters in human and mouse spermatozoa. Nat Struct Mol Biol 2010;6:679–687. 10.1038/nsmb.1821 20473313

[dmy021C30] Buenrostro JD, Giresi PG, Zaba LC, Chang HY, Greenleaf WJ Transposition of native chromatin for fast and sensitive epigenomic profiling of open chromatin, DNA-binding proteins and nucleosome position. Nat Methods 2013;12:1213–1218. 10.1038/nmeth.2688 PMC3959825 24097267

[dmy021C31] Buenrostro JD, Wu B, Litzenburger UM, Ruff D, Gonzales ML, Snyder MP, Chang HY, Greenleaf WJ Single-cell chromatin accessibility reveals principles of regulatory variation. Nature 2015;7561:486–490. 10.1038/nature14590 PMC4685948 26083756

[dmy021C32] Burton A, Torres-Padilla ME Chromatin dynamics in the regulation of cell fate allocation during early embryogenesis. Nat Rev Mol Cell Biol 2014;11:723–734. 10.1038/nrm3885 25303116

[dmy021C33] Canovas S, Ross PJ, Kelsey G, Coy P DNA methylation in embryo development: epigenetic impact of ART (Assisted Reproductive Technologies). Bioessays 2017;29: 1–11. 10.1002/bies.201700106 28940661

[dmy021C34] Carone BR, Hung JH, Hainer SJ, Chou MT, Carone DM, Weng Z, Fazzio TG, Rando OJ High-resolution mapping of chromatin packaging in mouse embryonic stem cells and sperm. Dev Cell 2014;1:11–22. 10.1016/j.devcel.2014.05.024 PMC4184102 24998598

[dmy021C35] Carrell DT Epigenetics of the male gamete. Fertil Steril 2012;2:267–274. 10.1016/j.fertnstert.2011.12.036 22289286

[dmy021C36] Cheng X Structural and functional coordination of DNA and histone methylation. Cold Spring Harb Perspect Biol 2014;8:a018747. 10.1101/cshperspect.a018747 PMC4107986 25085914

[dmy021C37] Choufani S, Shapiro JS, Susiarjo M, Butcher DT, Grafodatskaya D, Lou Y, Ferreira JC, Pinto D, Scherer SW, Shaffer LG et al A novel approach identifies new differentially methylated regions (DMRs) associated with imprinted genes. Genome Res 2011;3:465–476. 10.1101/gr.111922.110 PMC3044860 21324877

[dmy021C39] Clark SJ, Argelaguet R, Kapourani CA, Stubbs TM, Lee HJ, Alda-Catalinas C, Krueger F, Sanguinetti G, Kelsey G, Marioni JC et al scNMT-seq enables joint profiling of chromatin accessibility DNA methylation and transcription in single cells. Nat Commun 2018;1:781. 10.1038/s41467-018-03149-4 PMC5823944 29472610

[dmy021C40] Clouaire T, Webb S, Skene P, Illingworth R, Kerr A, Andrews R, Lee JH, Skalnik D, Bird A Cfp1 integrates both CpG content and gene activity for accurate H3K4me3 deposition in embryonic stem cells. Genes Dev 2012;15:1714–1728. 10.1101/gad.194209.112 PMC3418589 22855832

[dmy021C41] Cokus SJ, Feng S, Zhang X, Chen Z, Merriman B, Haudenschild CD, Pradhan S, Nelson SF, Pellegrini M, Jacobsen SE Shotgun bisulphite sequencing of the Arabidopsis genome reveals DNA methylation patterning. Nature 2008;7184:215–219. 10.1038/nature06745 PMC2377394 18278030

[dmy021C42] Court F, Tayama C, Romanelli V, Martin Trujillo A, Iglesias-Platas I, Okamura K, Sugahara N, Simon C, Moore H, Harness JV et al Genome-wide parent-of-origin DNA methylation analysis reveals the intricacies of human imprinting and suggests a germline methylation-independent mechanism of establishment. Genome Res 2014;24:554–569. 24402520 10.1101/gr.164913.113 PMC3975056

[dmy021C43] Crowley JJ, Zhabotynsky V, Sun W, Huang S, Pakatci IK, Kim Y, Wang JR, Morgan AP, Calaway JD, Aylor DL et al Analyses of allele-specific gene expression in highly divergent mouse crosses identifies pervasive allelic imbalance. Nat Genet 2015;4:353–360. 10.1038/ng.3222 PMC4380817 25730764

[dmy021C44] Cusanovich DA, Daza R, Adey A, Pliner HA, Christiansen L, Gunderson KL, Steemers FJ, Trapnell C, Shendure J Multiplex single cell profiling of chromatin accessibility by combinatorial cellular indexing. Science 2015;6237:910–914. 10.1126/science.aab1601 PMC4836442 25953818

[dmy021C45] Dahl JA, Jung I, Aanes H, Greggains GD, Manaf A, Lerdrup M, Li G, Kuan S, Li B, Lee AY et al Broad histone H3K4me3 domains in mouse oocytes modulate maternal-to-zygotic transition. Nature 2016;7621:548–552. 10.1038/nature19360 PMC6283663 27626377

[dmy021C46] Davies MJ, Moore VM, Willson KJ, Van Essen P, Priest K, Scott H, Haan EA, Chan A Reproductive technologies and the risk of birth defects. N Engl J Med 2012;19:1803–1813. 10.1056/NEJMoa1008095 22559061

[dmy021C47] Decato BE, Lopez-Tello J, Sferruzzi-Perri AN, Smith AD, Dean MD DNA methylation divergence and tissue specialization in the developing mouse placenta. Mol Biol Evol 2017;7:1702–1712. 10.1093/molbev/msx112 PMC6440273 28379409

[dmy021C48] Denomme MM, McCallie BR, Parks JC, Schoolcraft WB, Katz-Jaffe MG Alterations in the sperm histone-retained epigenome are associated with unexplained male factor infertility and poor blastocyst development in donor oocyte IVF cycles. Hum Reprod 2017;12:2443–2455. 10.1093/humrep/dex317 29087470

[dmy021C49] Dhayalan A, Rajavelu A, Rathert P, Tamas R, Jurkowska RZ, Ragozin S, Jeltsch A The Dnmt3a PWWP domain reads histone 3 lysine 36 trimethylation and guides DNA methylation. J Biol Chem 2010;34:26114–26120. 10.1074/jbc.M109.089433 PMC2924014 20547484

[dmy021C50] Dixon JR, Selvaraj S, Yue F, Kim A, Li Y, Shen Y, Hu M, Liu JS, Ren B Topological domains in mammalian genomes identified by analysis of chromatin interactions. Nature 2012;7398:376–380. 10.1038/nature11082 PMC3356448 22495300

[dmy021C51] Docherty LE, Rezwan FI, Poole RL, Turner CL, Kivuva E, Maher ER, Smithson SF, Hamilton-Shield JP, Patalan M, Gizewska M et al Mutations in NLRP5 are associated with reproductive wastage and multilocus imprinting disorders in humans. Nat Commun 2015;6:8086. 26323243 10.1038/ncomms9086 PMC4568303

[dmy021C52] Domcke S, Bardet AF, Adrian Ginno P, Hartl D, Burger L, Schubeler D Competition between DNA methylation and transcription factors determines binding of NRF1. Nature 2015;7583:575–579. 10.1038/nature16462 26675734

[dmy021C53] Dominguez-Salas P, Moore SE, Baker MS, Bergen AW, Cox SE, Dyer RA, Fulford AJ, Guan Y, Laritsky E, Silver MJ et al Maternal nutrition at conception modulates DNA methylation of human metastable epialleles. Nat Commun 2014;5:3746. 24781383 10.1038/ncomms4746 PMC4015319

[dmy021C54] Du J, Johnson LM, Jacobsen SE, Patel DJ DNA methylation pathways and their crosstalk with histone methylation. Nat Rev Mol Cell Biol 2015;9:519–532. 10.1038/nrm4043 PMC4672940 26296162

[dmy021C55] Du Z, Zheng H, Huang B, Ma R, Wu J, Zhang X, He J, Xiang Y, Wang Q, Li Y et al Allelic reprogramming of 3D chromatin architecture during early mammalian development. Nature 2017;7662:232–235. 10.1038/nature23263 28703188

[dmy021C56] Ehmcke J, Wistuba J, Schlatt S Spermatogonial stem cells: questions, models and perspectives. Hum Reprod Update 2006;3:275–282. 10.1093/humupd/dmk001 16446319

[dmy021C58] Erkek S, Hisano M, Liang CY, Gill M, Murr R, Dieker J, Schubeler D, van der Vlag J, Stadler MB, Peters AH Molecular determinants of nucleosome retention at CpG-rich sequences in mouse spermatozoa. Nat Struct Mol Biol 2013;7:868–875. 10.1038/nsmb.2599 23770822

[dmy021C59] Ernst P, Fisher JK, Avery W, Wade S, Foy D, Korsmeyer SJ Definitive hematopoiesis requires the mixed-lineage leukemia gene. Dev Cell 2004;3:437–443. 10.1016/s1534-5807(04)00061-9 15030765

[dmy021C60] Fauser BC, Devroey P, Diedrich K, Balaban B, Bonduelle M, Delemarre-van de Waal HA, Estella C, Ezcurra D, Geraedts JP, Howles CM et al. Health outcomes of children born after IVF/ICSI: a review of current expert opinion and literature. Reprod Biomed Online 2014;2:162–182. 10.1016/j.rbmo.2013.10.013 24365026

[dmy021C61] Flyamer IM, Gassler J, Imakaev M, Brandao HB, Ulianov SV, Abdennur N, Razin SV, Mirny LA, Tachibana-Konwalski K Single-nucleus Hi-C reveals unique chromatin reorganization at oocyte-to-zygote transition. Nature 2017;7648:110–114. 10.1038/nature21711 PMC5639698 28355183

[dmy021C62] Fogarty NME, McCarthy A, Snijders KE, Powell BE, Kubikova N, Blakeley P, Lea R, Elder K, Wamaitha SE, Kim D et al Genome editing reveals a role for OCT4 in human embryogenesis. Nature 2017;7674:67–73. 10.1038/nature24033 PMC5815497 28953884

[dmy021C63] Frost JM, Moore GE The importance of imprinting in the human placenta. PLoS Genet 2010;7:e1001015. 10.1371/journal.pgen.1001015 PMC2895656 20617174

[dmy021C64] Frost JM, Udayashankar R, Moore HD, Moore GE Telomeric NAP1L4 and OSBPL5 of the KCNQ1 cluster, and the DECORIN gene are not imprinted in human trophoblast stem cells. PLoS One 2010;7:e11595. 10.1371/journal.pone.0011595 PMC2904374 20644730

[dmy021C65] Furlan G, Rougeulle C Function and evolution of the long noncoding RNA circuitry orchestrating X-chromosome inactivation in mammals. Wiley Interdiscip Rev RNA 2016;5:702–722. 10.1002/wrna.1359 27173581

[dmy021C66] Gahurova L, Tomizawa SI, Smallwood SA, Stewart-Morgan KR, Saadeh H, Kim J, Andrews SR, Chen T, Kelsey G Transcription and chromatin determinants of de novo DNA methylation timing in oocytes. Epigenetics Chromatin 2017;10:25. 28507606 10.1186/s13072-017-0133-5 PMC5429541

[dmy021C68] Garcia-Peiro A, Martinez-Heredia J, Oliver-Bonet M, Abad C, Amengual MJ, Navarro J, Jones C, Coward K, Gosalvez J, Benet J Protamine 1 to protamine 2 ratio correlates with dynamic aspects of DNA fragmentation in human sperm. Fertil Steril 2011;1:105–109. 10.1016/j.fertnstert.2010.06.053 20667534

[dmy021C69] Garg P, Borel C, Sharp AJ Detection of parent-of-origin specific expression quantitative trait loci by cis-association analysis of gene expression in trios. PLoS One 2012;8:e41695. 10.1371/journal.pone.0041695 PMC3422236 22912676

[dmy021C70] Geraghty AA, Lindsay KL, Alberdi G, McAuliffe FM, Gibney ER Nutrition during pregnancy impacts offspring’s epigenetic status-evidence from human and animal studies. Nutr Metab Insights 2016;8:41–47. 26917970 10.4137/NMI.S29527 PMC4758803

[dmy021C71] Gkountela S, Zhang KX, Shafiq TA, Liao WW, Hargan-Calvopina J, Chen PY, Clark AT DNA demethylation dynamics in the human prenatal germline. Cell 2015;6:1425–1436. 10.1016/j.cell.2015.05.012 PMC4458157 26004067

[dmy021C72] Glaser S, Schaft J, Lubitz S, Vintersten K, van der Hoeven F, Tufteland KR, Aasland R, Anastassiadis K, Ang SL, Stewart AF Multiple epigenetic maintenance factors implicated by the loss of Mll2 in mouse development. Development 2006;8:1423–1432. 10.1242/dev.02302 16540515

[dmy021C73] Gougeon A Dynamics of follicular growth in the human: a model from preliminary results. Hum Reprod 1986;2:81–87. 10.1093/oxfordjournals.humrep.a136365 3558758

[dmy021C74] Gu TP, Guo F, Yang H, Wu HP, Xu GF, Liu W, Xie ZG, Shi L, He X, Jin SG et al The role of Tet3 DNA dioxygenase in epigenetic reprogramming by oocytes. Nature 2011;7366:606–610. 10.1038/nature10443 21892189

[dmy021C75] Guibert S, Forne T, Weber M Global profiling of DNA methylation erasure in mouse primordial germ cells. Genome Res 2012;4:633–641. 10.1101/gr.130997.111 PMC3317146 22357612

[dmy021C76] Guo H, Hu B, Yan L, Yong J, Wu Y, Gao Y, Guo F, Hou Y, Fan X, Dong J et al DNA methylation and chromatin accessibility profiling of mouse and human fetal germ cells. Cell Res 2017b;2:165–183. 10.1038/cr.2016.128 PMC5339845 27824029

[dmy021C77] Guo F, Li L, Li J, Wu X, Hu B, Zhu P, Wen L, Tang F Single-cell multi-omics sequencing of mouse early embryos and embryonic stem cells. Cell Res 2017a;8:967–988. 10.1038/cr.2017.82 PMC5539349 28621329

[dmy021C78] Guo F, Yan L, Guo H, Li L, Hu B, Zhao Y, Yong J, Hu Y, Wang X, Wei Y et al The transcriptome and DNA methylome landscapes of human primordial germ cells. Cell 2015;6:1437–1452. 10.1016/j.cell.2015.05.015 26046443

[dmy021C79] Guo H, Zhu P, Wu X, Li X, Wen L, Tang F Single-cell methylome landscapes of mouse embryonic stem cells and early embryos analyzed using reduced representation bisulfite sequencing. Genome Res 2013;12:2126–2135. 10.1101/gr.161679.113 PMC3847781 24179143

[dmy021C80] Guo H, Zhu P, Yan L, Li R, Hu B, Lian Y, Yan J, Ren X, Lin S, Li J et al The DNA methylation landscape of human early embryos. Nature 2014;7511:606–610. 10.1038/nature13544 25079557

[dmy021C81] Hajkova P, Erhardt S, Lane N, Haaf T, El-Maarri O, Reik W, Walter J, Surani MA Epigenetic reprogramming in mouse primordial germ cells. Mech Dev 2002;1-2:15–23. 10.1016/s0925-4773(02)00181-8 12204247

[dmy021C82] Hamada H, Okae H, Toh H, Chiba H, Hiura H, Shirane K, Sato T, Suyama M, Yaegashi N, Sasaki H et al Allele-specific methylome and transcriptome analysis reveals widespread imprinting in the human placenta. Am J Hum Genet 2016;5:1045–1058. 10.1016/j.ajhg.2016.08.021 PMC5097938 27843122

[dmy021C83] Hammoud SS, Nix DA, Hammoud AO, Gibson M, Cairns BR, Carrell DT Genome-wide analysis identifies changes in histone retention and epigenetic modifications at developmental and imprinted gene loci in the sperm of infertile men. Hum Reprod 2011;9:2558–2569. 10.1093/humrep/der192 PMC3157626 21685136

[dmy021C84] Hammoud SS, Nix DA, Zhang H, Purwar J, Carrell DT, Cairns BR Distinctive chromatin in human sperm packages genes for embryo development. Nature 2009;7254:473–478. 10.1038/nature08162 PMC2858064 19525931

[dmy021C85] Hanna CW, Kelsey G The specification of imprints in mammals. Heredity (Edinb) 2014;2:176–183. 10.1038/hdy.2014.54 PMC4105455 24939713

[dmy021C86] Hanna CW, McFadden DE, Robinson WP DNA methylation profiling of placental villi from karyotypically normal miscarriage and recurrent miscarriage. Am J Pathol 2013;6:2276–2284. 10.1016/j.ajpath.2013.02.021 23583422

[dmy021C87] Hanna CW, Penaherrera MS, Saadeh H, Andrews S, McFadden DE, Kelsey G, Robinson WP Pervasive polymorphic imprinted methylation in the human placenta. Genome Res 2016;6:756–767. 10.1101/gr.196139.115 PMC4889973 26769960

[dmy021C88] Hanna CW, Taudt A, Huang J, Gahurova L, Kranz A, Andrews S, Dean W, Stewart AF, Colome-Tatche M, Kelsey G MLL2 conveys transcription-independent H3K4 trimethylation in oocytes. Nat Struct Mol Biol 2018;1:73–82. 10.1038/s41594-017-0013-5 29323282

[dmy021C89] Hart R, Norman RJ The longer-term health outcomes for children born as a result of IVF treatment: Part I – General health outcomes. Hum Reprod Update 2013;3:232–243. 10.1093/humupd/dms062 23449642

[dmy021C90] Hendrich B, Bird A Identification and characterization of a family of mammalian methyl-CpG binding proteins. Mol Cell Biol 1998;11:6538–6547. 10.1128/mcb.18.11.6538 PMC109239 9774669

[dmy021C91] Hill PW, Amouroux R, Hajkova P DNA demethylation, Tet proteins and 5-hydroxymethylcytosine in epigenetic reprogramming: an emerging complex story. Genomics 2014;5:324–333. 10.1016/j.ygeno.2014.08.012 25173569

[dmy021C92] Hiura H, Obata Y, Komiyama J, Shirai M, Kono T Oocyte growth-dependent progression of maternal imprinting in mice. Genes Cells 2006;4:353–361. 10.1111/j.1365-2443.2006.00943.x 16611239

[dmy021C93] Hiura H, Okae H, Chiba H, Miyauchi N, Sato F, Sato A, Arima T Imprinting methylation errors in ART. Reprod Med Biol 2014;4:193–202. 10.1007/s12522-014-0183-3 PMC4182590 25298744

[dmy021C94] Howe FS, Fischl H, Murray SC, Mellor J Is H3K4me3 instructive for transcription activation? Bioessays 2017;1:1–12. 10.1002/bies.201600095 28004446

[dmy021C95] Inoue A, Jiang L, Lu F, Suzuki T, Zhang Y Maternal H3K27me3 controls DNA methylation-independent imprinting. Nature 2017a;7664:419–424. 10.1038/nature23262 PMC9674007 28723896

[dmy021C96] Inoue A, Jiang L, Lu F, Zhang Y Genomic imprinting of Xist by maternal H3K27me3. Genes Dev 2017b;19:1927–1932. 10.1101/gad.304113.117 PMC5710138 29089420

[dmy021C97] Inoue A, Zhang Y Replication-dependent loss of 5-hydroxymethylcytosine in mouse preimplantation embryos. Science 2011;6053:194. 10.1126/science.1212483 PMC3799877 21940858

[dmy021C98] Ishida M, Moore GE The role of imprinted genes in humans. Mol Aspects Med 2013;4:826–840. 10.1016/j.mam.2012.06.009 22771538

[dmy021C99] Januar V, Desoye G, Novakovic B, Cvitic S, Saffery R Epigenetic regulation of human placental function and pregnancy outcome: considerations for causal inference. Am J Obstet Gynecol 2015;4:S182–S196. 10.1016/j.ajog.2015.07.011 26428498

[dmy021C100] Jin W, Tang Q, Wan M, Cui K, Zhang Y, Ren G, Ni B, Sklar J, Przytycka TM, Childs R et al Genome-wide detection of DNase I hypersensitive sites in single cells and FFPE tissue samples. Nature 2015;7580:142–146. 10.1038/nature15740 PMC4697938 26605532

[dmy021C101] Jones PA Functions of DNA methylation: islands, start sites, gene bodies and beyond. Nat Rev Genet 2012;7:484–492. 10.1038/nrg3230 22641018

[dmy021C102] Joubert BR, Haberg SE, Nilsen RM, Wang X, Vollset SE, Murphy SK, Huang Z, Hoyo C, Midttun O, Cupul-Uicab LA et al 450K epigenome-wide scan identifies differential DNA methylation in newborns related to maternal smoking during pregnancy. Environ Health Perspect 2012;10:1425–1431. 10.1289/ehp.1205412 PMC3491949 22851337

[dmy021C103] Kagiwada S, Kurimoto K, Hirota T, Yamaji M, Saitou M Replication-coupled passive DNA demethylation for the erasure of genome imprints in mice. EMBO J 2013;3:340–353. 10.1038/emboj.2012.331 PMC3567490 23241950

[dmy021C104] Kajii T, Ohama K Androgenetic origin of hydatidiform mole. Nature 1977;5621:633–634. 10.1038/268633a0 561314

[dmy021C105] Kaneda M, Okano M, Hata K, Sado T, Tsujimoto N, Li E, Sasaki H Essential role for de novo DNA methyltransferase Dnmt3a in paternal and maternal imprinting. Nature 2004;6994:900–903. 10.1038/nature02633 15215868

[dmy021C106] Ke Y, Xu Y, Chen X, Feng S, Liu Z, Sun Y, Yao X, Li F, Zhu W, Gao L et al 3D chromatin structures of mature gametes and structural reprogramming during mammalian embryogenesis. Cell 2017;2:367–381.e20. 10.1016/j.cell.2017.06.029 28709003

[dmy021C107] Kelly TK, Liu Y, Lay FD, Liang G, Berman BP, Jones PA Genome-wide mapping of nucleosome positioning and DNA methylation within individual DNA molecules. Genome Res 2012;12:2497–2506. 10.1101/gr.143008.112 PMC3514679 22960375

[dmy021C108] Kelsey G, Stegle O, Reik W Single-cell epigenomics: recording the past and predicting the future. Science 2017;6359:69–75. 10.1126/science.aan6826 28983045

[dmy021C109] Kobayashi H, Hiura H, John RM, Sato A, Otsu E, Kobayashi N, Suzuki R, Suzuki F, Hayashi C, Utsunomiya T et al DNA methylation errors at imprinted loci after assisted conception originate in the parental sperm. Eur J Hum Genet 2009;12:1582–1591. 10.1038/ejhg.2009.68 PMC2845511 19471309

[dmy021C110] Kobayashi H, Sakurai T, Imai M, Takahashi N, Fukuda A, Yayoi O, Sato S, Nakabayashi K, Hata K, Sotomaru Y et al Contribution of intragenic DNA methylation in mouse gametic DNA methylomes to establish oocyte-specific heritable marks. PLoS Genet 2012;1:e1002440. 10.1371/journal.pgen.1002440 PMC3252278 22242016

[dmy021C111] Koukoura O, Sifakis S, Spandidos DA DNA methylation in the human placenta and fetal growth (review). Mol Med Rep 2012;4:883–889. 10.3892/mmr.2012.763 PMC3493070 22294146

[dmy021C112] Kupers LK, Xu X, Jankipersadsing SA, Vaez A, la Bastide-van Gemert S, Scholtens S, Nolte IM, Richmond CL, Felix JF et al. DNA methylation mediates the effect of maternal smoking during pregnancy on birthweight of the offspring Int J Epidemiol. 2015;4:1224–1237. 10.1093/ije/dyv048 PMC4588868 25862628

[dmy021C113] Lachner M, O’Carroll D, Rea S, Mechtler K, Jenuwein T Methylation of histone H3 lysine 9 creates a binding site for HP1 proteins. Nature 2001;6824:116–120. 10.1038/35065132 11242053

[dmy021C114] Lewis A, Green K, Dawson C, Redrup L, Huynh KD, Lee JT, Hemberger M, Reik W Epigenetic dynamics of the Kcnq1 imprinted domain in the early embryo. Development 2006;21:4203–4210. 10.1242/dev.02612 17021040

[dmy021C115] Lewis A, Mitsuya K, Umlauf D, Smith P, Dean W, Walter J, Higgins M, Feil R, Reik W Imprinting on distal chromosome 7 in the placenta involves repressive histone methylation independent of DNA methylation. Nat Genet 2004;12:1291–1295. 10.1038/ng1468 15516931

[dmy021C116] Li E, Bestor TH, Jaenisch R Targeted mutation of the DNA methyltransferase gene results in embryonic lethality. Cell 1992;6:915–926. 10.1016/0092-8674(92)90611-f 1606615

[dmy021C117] Li X, Ito M, Zhou F, Youngson N, Zuo X, Leder P, Ferguson-Smith AC A maternal-zygotic effect gene, Zfp57, maintains both maternal and paternal imprints. Dev Cell 2008;4:547–557. 10.1016/j.devcel.2008.08.014 PMC2593089 18854139

[dmy021C118] Lieberman-Aiden E, van Berkum NL, Williams L, Imakaev M, Ragoczy T, Telling A, Amit I, Lajoie BR, Sabo PJ, Dorschner MO et al Comprehensive mapping of long-range interactions reveals folding principles of the human genome. Science 2009;5950:289–293. 10.1126/science.1181369 PMC2858594 19815776

[dmy021C119] Liu X, Wang C, Liu W, Li J, Li C, Kou X, Chen J, Zhao Y, Gao H, Wang H et al Distinct features of H3K4me3 and H3K27me3 chromatin domains in pre-implantation embryos. Nature 2016;7621:558–562. 10.1038/nature19362 27626379

[dmy021C120] Liu H, Zhang Y, Gu HT, Feng QL, Liu JY, Zhou J, Yan F Association between assisted reproductive technology and cardiac alteration at age 5 years. JAMA Pediatr 2015;6:603–605. 10.1001/jamapediatrics.2015.0214 25915111

[dmy021C121] Lu F, Liu Y, Inoue A, Suzuki T, Zhao K, Zhang Y Establishing chromatin regulatory landscape during mouse preimplantation development. Cell 2016;6:1375–1388. 10.1016/j.cell.2016.05.050 PMC6625655 27259149

[dmy021C122] Mackay DJ, Callaway JL, Marks SM, White HE, Acerini CL, Boonen SE, Dayanikli P, Firth HV, Goodship JA, Haemers AP et al Hypomethylation of multiple imprinted loci in individuals with transient neonatal diabetes is associated with mutations in ZFP57. Nat Genet 2008;8:949–951. 10.1038/ng.187 18622393

[dmy021C123] Maenohara S, Unoki M, Toh H, Ohishi H, Sharif J, Koseki H, Sasaki H Role of UHRF1 in de novo DNA methylation in oocytes and maintenance methylation in preimplantation embryos. PLoS Genet 2017;10:e1007042. 10.1371/journal.pgen.1007042 PMC5643148 28976982

[dmy021C124] Manzo M, Wirz J, Ambrosi C, Villasenor R, Roschitzki B, Baubec T Isoform-specific localization of DNMT3A regulates DNA methylation fidelity at bivalent CpG islands. EMBO J 2017;36:3421–3434. 29074627 10.15252/embj.201797038 PMC5709737

[dmy021C125] Margaritis T, Oreal V, Brabers N, Maestroni L, Vitaliano-Prunier A, Benschop JJ, van Hooff S, van Leenen D, Dargemont C, Geli V et al Two distinct repressive mechanisms for histone 3 lysine 4 methylation through promoting 3'-end antisense transcription. PLoS Genet 2012;9:e1002952. 10.1371/journal.pgen.1002952 PMC3447963 23028359

[dmy021C126] Marques CJ, Joao Pinho M, Carvalho F, Bieche I, Barros A, Sousa M DNA methylation imprinting marks and DNA methyltransferase expression in human spermatogenic cell stages. Epigenetics 2011;11:1354–1361. 10.4161/epi.6.11.17993 22048249

[dmy021C127] Mattson BA, Albertini DF Oogenesis: chromatin and microtubule dynamics during meiotic prophase. Mol Reprod Dev 1990;4:374–383. 10.1002/mrd.1080250411 1691651

[dmy021C128] McGrath J, Solter D Completion of mouse embryogenesis requires both the maternal and paternal genomes. Cell 1984;1:179–183. 10.1016/0092-8674(84)90313-1 6722870

[dmy021C129] Messerschmidt DM Should I stay or should I go: protection and maintenance of DNA methylation at imprinted genes. Epigenetics 2012;9:969–975. 10.4161/epi.21337 PMC3515016 22869105

[dmy021C130] Messerschmidt DM, de Vries W, Ito M, Solter D, Ferguson-Smith A, Knowles BB Trim28 is required for epigenetic stability during mouse oocyte to embryo transition. Science 2012;6075:1499–1502. 10.1126/science.1216154 22442485

[dmy021C131] Metsalu T, Viltrop T, Tiirats A, Rajashekar B, Reimann E, Koks S, Rull K, Milani L, Acharya G, Basnet P et al Using RNA sequencing for identifying gene imprinting and random monoallelic expression in human placenta. Epigenetics 2014;10:1397–1409. 10.4161/15592294.2014.970052 PMC4623103 25437054

[dmy021C132] Migeon BR, Do TT In search of non-random X inactivation: studies of fetal membranes heterozygous for glucose-6-phosphate dehydrogenase. Am J Hum Genet 1979;5:581–585. PMC1685908 507052

[dmy021C133] Mikkelsen TS, Ku M, Jaffe DB, Issac B, Lieberman E, Giannoukos G, Alvarez P, Brockman W, Kim TK, Koche RP et al Genome-wide maps of chromatin state in pluripotent and lineage-committed cells. Nature 2007;7153:553–560. 10.1038/nature06008 PMC2921165 17603471

[dmy021C134] Miller D, Brinkworth M, Iles D Paternal DNA packaging in spermatozoa: more than the sum of its parts? DNA, histones, protamines and epigenetics. Reproduction 2010;2:287–301. 10.1530/REP-09-0281 19759174

[dmy021C135] Minoux M, Holwerda S, Vitobello A, Kitazawa T, Kohler H, Stadler MB, Rijli FM Gene bivalency at Polycomb domains regulates cranial neural crest positional identity. Science 2017;6332:eaal2913. 10.1126/science.aal2913 28360266

[dmy021C136] Miura F, Enomoto Y, Dairiki R, Ito T Amplification-free whole-genome bisulfite sequencing by post-bisulfite adaptor tagging. Nucleic Acids Res 2012;17:e136. 10.1093/nar/gks454 PMC3458524 22649061

[dmy021C137] Molaro A, Hodges E, Fang F, Song Q, McCombie WR, Hannon GJ, Smith AD Sperm methylation profiles reveal features of epigenetic inheritance and evolution in primates. Cell 2011;6:1029–1041. 10.1016/j.cell.2011.08.016 PMC3205962 21925323

[dmy021C138] Monk D, Sanchez-Delgado M, Fisher R NLRPs, the subcortical maternal complex and genomic imprinting. Reproduction 2017;6:R161–R170. 10.1530/REP-17-0465 28916717

[dmy021C139] Montjean D, Zini A, Ravel C, Belloc S, Dalleac A, Copin H, Boyer P, McElreavey K, Benkhalifa M Sperm global DNA methylation level: association with semen parameters and genome integrity. Andrology 2015;2:235–240. 10.1111/andr.12001 25755112

[dmy021C140] Moore GE, Ishida M, Demetriou C, Al-Olabi L, Leon LJ, Thomas AC, Abu-Amero S, Frost JM, Stafford JL, Chaoqun Y et al The role and interaction of imprinted genes in human fetal growth. Philos Trans R Soc Lond B Biol Sci 2015;1663:20140074. 10.1098/rstb.2014.0074 PMC4305174 25602077

[dmy021C141] Murdoch S, Djuric U, Mazhar B, Seoud M, Khan R, Kuick R, Bagga R, Kircheisen R, Ao A, Ratti B et al Mutations in NALP7 cause recurrent hydatidiform moles and reproductive wastage in humans. Nat Genet 2006;3:300–302. 10.1038/ng1740 16462743

[dmy021C142] Nagano T, Lubling Y, Stevens TJ, Schoenfelder S, Yaffe E, Dean W, Laue ED, Tanay A, Fraser P Single-cell Hi-C reveals cell-to-cell variability in chromosome structure. Nature 2013;7469:59–64. 10.1038/nature12593 PMC3869051 24067610

[dmy021C143] Nakamura T, Arai Y, Umehara H, Masuhara M, Kimura T, Taniguchi H, Sekimoto T, Ikawa M, Yoneda Y, Okabe M et al PGC7/Stella protects against DNA demethylation in early embryogenesis. Nat Cell Biol 2007;1:64–71. 10.1038/ncb1519 17143267

[dmy021C144] Nakamura T, Liu YJ, Nakashima H, Umehara H, Inoue K, Matoba S, Tachibana M, Ogura A, Shinkai Y, Nakano T PGC7 binds histone H3K9me2 to protect against conversion of 5mC to 5hmC in early embryos. Nature 2012;7403:415–419. 10.1038/nature11093 22722204

[dmy021C145] Neri F, Rapelli S, Krepelova A, Incarnato D, Parlato C, Basile G, Maldotti M, Anselmi F, Oliviero S Intragenic DNA methylation prevents spurious transcription initiation. Nature 2017;7643:72–77. 10.1038/nature21373 28225755

[dmy021C146] Ng RK, Dean W, Dawson C, Lucifero D, Madeja Z, Reik W, Hemberger M Epigenetic restriction of embryonic cell lineage fate by methylation of Elf5. Nat Cell Biol 2008;11:1280–1290. 10.1038/ncb1786 PMC2635539 18836439

[dmy021C147] Noguer-Dance M, Abu-Amero S, Al-Khtib M, Lefevre A, Coullin P, Moore GE, Cavaille J The primate-specific microRNA gene cluster (C19MC) is imprinted in the placenta. Hum Mol Genet 2010;18:3566–3582. 10.1093/hmg/ddq272 20610438

[dmy021C148] Novakovic B, Ryan J, Pereira N, Boughton B, Craig JM, Saffery R Postnatal stability, tissue, and time specific effects of AHRR methylation change in response to maternal smoking in pregnancy. Epigenetics 2014;3:377–386. 10.4161/epi.27248 PMC4053456 24270552

[dmy021C149] O’Carroll D, Erhardt S, Pagani M, Barton SC, Surani MA, Jenuwein T The polycomb-group gene Ezh2 is required for early mouse development. Mol Cell Biol 2001;13:4330–4336. 10.1128/MCB.21.13.4330-4336.2001 PMC87093 11390661

[dmy021C150] Oakes CC, La Salle S, Smiraglia DJ, Robaire B, Trasler JM Developmental acquisition of genome-wide DNA methylation occurs prior to meiosis in male germ cells. Dev Biol 2007;2:368–379. 10.1016/j.ydbio.2007.05.002 17559830

[dmy021C151] Okae H, Chiba H, Hiura H, Hamada H, Sato A, Utsunomiya T, Kikuchi H, Yoshida H, Tanaka A, Suyama M et al Genome-wide analysis of DNA methylation dynamics during early human development. PLoS Genet 2014;12:e1004868. 10.1371/journal.pgen.1004868 PMC4263407 25501653

[dmy021C152] Okano M, Bell DW, Haber DA, Li E DNA methyltransferases Dnmt3a and Dnmt3b are essential for de novo methylation and mammalian development. Cell 1999;3:247–257. 10.1016/s0092-8674(00)81656-6 10555141

[dmy021C153] Ooi SK, Qiu C, Bernstein E, Li K, Jia D, Yang Z, Erdjument-Bromage H, Tempst P, Lin SP, Allis CD et al DNMT3L connects unmethylated lysine 4 of histone H3 to de novo methylation of DNA. Nature 2007;7154:714–717. 10.1038/nature05987 PMC2650820 17687327

[dmy021C154] Palma-Gudiel H, Cordova-Palomera A, Eixarch E, Deuschle M, Fananas L Maternal psychosocial stress during pregnancy alters the epigenetic signature of the glucocorticoid receptor gene promoter in their offspring: a meta-analysis. Epigenetics 2015;10:893–902. 26327302 10.1080/15592294.2015.1088630 PMC4844196

[dmy021C155] Parry DA, Logan CV, Hayward BE, Shires M, Landolsi H, Diggle C, Carr I, Rittore C, Touitou I, Philibert L et al Mutations causing familial biparental hydatidiform mole implicate c6orf221 as a possible regulator of genomic imprinting in the human oocyte. Am J Hum Genet 2011;3:451–458. 10.1016/j.ajhg.2011.08.002 PMC3169823 21885028

[dmy021C156] Penaherrera MS, Ma S, Ho Yuen B, Brown CJ, Robinson WP X-chromosome inactivation (XCI) patterns in placental tissues of a paternally derived bal t(X;20) case. Am J Med Genet A 2003;1:29–34. 10.1002/ajmg.a.10041 12605437

[dmy021C157] Peters J The role of genomic imprinting in biology and disease: an expanding view. Nat Rev Genet 2014;8:517–530. 10.1038/nrg3766 24958438

[dmy021C158] Pliushch G, Schneider E, Weise D, El Hajj N, Tresch A, Seidmann L, Coerdt W, Muller AM, Zechner U, Haaf T Extreme methylation values of imprinted genes in human abortions and stillbirths. Am J Pathol 2010;3:1084–1090. 10.2353/ajpath.2010.090764 PMC2832130 20093482

[dmy021C159] Pott S Simultaneous measurement of chromatin accessibility, DNA methylation, and nucleosome phasing in single cells. Elife 2017;6:e23203. 28653622 10.7554/eLife.23203 PMC5487215

[dmy021C160] Puschendorf M, Terranova R, Boutsma E, Mao X, Isono K, Brykczynska U, Kolb C, Otte AP, Koseki H, Orkin SH et al PRC1 and Suv39h specify parental asymmetry at constitutive heterochromatin in early mouse embryos. Nat Genet 2008;4:411–420. 10.1038/ng.99 18311137

[dmy021C161] Quenneville S, Verde G, Corsinotti A, Kapopoulou A, Jakobsson J, Offner S, Baglivo I, Pedone PV, Grimaldi G, Riccio A et al In embryonic stem cells, ZFP57/KAP1 recognize a methylated hexanucleotide to affect chromatin and DNA methylation of imprinting control regions. Mol Cell 2011;3:361–372. 10.1016/j.molcel.2011.08.032 PMC3210328 22055183

[dmy021C162] Reik W, Walter J Genomic imprinting: parental influence on the genome. Nat Rev Genet 2001;1:21–32. 10.1038/35047554 11253064

[dmy021C163] Robertson G, Hirst M, Bainbridge M, Bilenky M, Zhao Y, Zeng T, Euskirchen G, Bernier B, Varhol R, Delaney A et al Genome-wide profiles of STAT1 DNA association using chromatin immunoprecipitation and massively parallel sequencing. Nat Methods 2007;8:651–657. 10.1038/nmeth1068 17558387

[dmy021C164] Robinson WP, Price EM The human placental methylome. Cold Spring Harb Perspect Med 2015;5:a023044. 25722473 10.1101/cshperspect.a023044 PMC4448590

[dmy021C165] Roseboom T, de Rooij S, Painter R The Dutch famine and its long-term consequences for adult health. Early Hum Dev 2006;8:485–491. 10.1016/j.earlhumdev.2006.07.001 16876341

[dmy021C166] Rossant J, Sanford JP, Chapman VM, Andrews GK Undermethylation of structural gene sequences in extraembryonic lineages of the mouse. Dev Biol 1986;2:567–573. 10.1016/0012-1606(86)90325-8 2428685

[dmy021C167] Rotem A, Ram O, Shoresh N, Sperling RA, Goren A, Weitz DA, Bernstein BE Single-cell ChIP-seq reveals cell subpopulations defined by chromatin state. Nat Biotechnol 2015;11:1165–1172. 10.1038/nbt.3383 PMC4636926 26458175

[dmy021C168] Rothbart SB, Strahl BD Interpreting the language of histone and DNA modifications. Biochim Biophys Acta 2014;8:627–643. 10.1016/j.bbagrm.2014.03.001 PMC4099259 24631868

[dmy021C169] Rubin AJ, Barajas BC, Furlan-Magaril M, Lopez-Pajares V, Mumbach MR, Howard I, Kim DS, Boxer LD, Cairns J, Spivakov M et al Lineage-specific dynamic and pre-established enhancer-promoter contacts cooperate in terminal differentiation. Nat Genet 2017;10:1522–1528. 10.1038/ng.3935 PMC5715812 28805829

[dmy021C170] Rugg-Gunn PJ, Cox BJ, Ralston A, Rossant J Distinct histone modifications in stem cell lines and tissue lineages from the early mouse embryo. Proc Natl Acad Sci U S A 2010;24:10783–10790. 10.1073/pnas.0914507107 PMC2890770 20479220

[dmy021C171] Sanchez-Delgado M, Court F, Vidal E, Medrano J, Monteagudo-Sanchez A, Martin-Trujillo A, Tayama C, Iglesias-Platas I, Kondova I, Bontrop R et al Human oocyte-derived methylation differences persist in the placenta revealing widespread transient imprinting. PLoS Genet 2016a;11:e1006427. 10.1371/journal.pgen.1006427 PMC5106035 27835649

[dmy021C172] Sanchez-Delgado M, Riccio A, Eggermann T, Maher ER, Lapunzina P, Mackay D, Monk D Causes and consequences of multi-locus imprinting disturbances in humans. Trends Genet 2016b;7:444–455. 10.1016/j.tig.2016.05.001 27235113

[dmy021C173] Santoni FA, Stamoulis G, Garieri M, Falconnet E, Ribaux P, Borel C, Antonarakis SE Detection of imprinted genes by single-cell allele-specific gene expression. Am J Hum Genet 2017;3:444–453. 10.1016/j.ajhg.2017.01.028 PMC5339288 28190458

[dmy021C174] Santos F, Hendrich B, Reik W, Dean W Dynamic reprogramming of DNA methylation in the early mouse embryo. Dev Biol 2002;1:172–182. 10.1006/dbio.2001.0501 11784103

[dmy021C175] Schroeder DI, Blair JD, Lott P, Yu HO, Hong D, Crary F, Ashwood P, Walker C, Korf I, Robinson WP et al The human placenta methylome. Proc Natl Acad Sci USA 2013;15:6037–6042. 10.1073/pnas.1215145110 PMC3625261 23530188

[dmy021C176] Seisenberger S, Andrews S, Krueger F, Arand J, Walter J, Santos F, Popp C, Thienpont B, Dean W, Reik W The dynamics of genome-wide DNA methylation reprogramming in mouse primordial germ cells. Mol Cell 2012;6:849–862. 10.1016/j.molcel.2012.11.001 PMC3533687 23219530

[dmy021C177] Seki Y, Hayashi K, Itoh K, Mizugaki M, Saitou M, Matsui Y Extensive and orderly reprogramming of genome-wide chromatin modifications associated with specification and early development of germ cells in mice. Dev Biol 2005;2:440–458. 10.1016/j.ydbio.2004.11.025 15680362

[dmy021C178] Shahbazi MN, Jedrusik A, Vuoristo S, Recher G, Hupalowska A, Bolton V, Fogarty NNM, Campbell A, Devito L, Ilic D et al Self-organization of the human embryo in the absence of maternal tissues. Nat Cell Biol 2016;6:700–708. 10.1038/ncb3347 PMC5049689 27144686

[dmy021C179] Sharif J, Muto M, Takebayashi S, Suetake I, Iwamatsu A, Endo TA, Shinga J, Mizutani-Koseki Y, Toyoda T, Okamura K et al The SRA protein Np95 mediates epigenetic inheritance by recruiting Dnmt1 to methylated DNA. Nature 2007;7171:908–912. 10.1038/nature06397 17994007

[dmy021C180] Sharp GC, Arathimos R, Reese SE, Page CM, Felix J, Kupers LK, Rifas-Shiman SL, Liu C Cohorts for Heart and Aging Research in Genomic Epidemiology plus (CHARGE +) methylation alcohol working group, Burrows K *et al.* Maternal alcohol consumption and offspring DNA methylation: findings from six general population-based birth cohorts. Epigenomics 2018;1:27–42. 10.2217/epi-2017-0095 PMC5753623 29172695

[dmy021C181] Shirane K, Toh H, Kobayashi H, Miura F, Chiba H, Ito T, Kono T, Sasaki H Mouse oocyte methylomes at base resolution reveal genome-wide accumulation of non-CpG methylation and role of DNA methyltransferases. PLoS Genet 2013;4:e1003439. 10.1371/journal.pgen.1003439 PMC3630097 23637617

[dmy021C182] Siklenka K, Erkek S, Godmann M, Lambrot R, McGraw S, Lafleur C, Cohen T, Xia J, Suderman M, Hallett M et al Disruption of histone methylation in developing sperm impairs offspring health transgenerationally. Science 2015;6261:aab2006. 10.1126/science.aab2006 26449473

[dmy021C183] Skene PJ, Henikoff JG, Henikoff S Targeted in situ genome-wide profiling with high efficiency for low cell numbers. Nat Protoc 2018;5:1006–1019. 10.1038/nprot.2018.015 29651053

[dmy021C184] Smallwood SA, Kelsey G Genome-wide analysis of DNA methylation in low cell numbers by reduced representation bisulfite sequencing. Methods Mol Biol 2012;925:187–197. 22907498 10.1007/978-1-62703-011-3_12

[dmy021C185] Smallwood SA, Lee HJ, Angermueller C, Krueger F, Saadeh H, Peat J, Andrews SR, Stegle O, Reik W, Kelsey G Single-cell genome-wide bisulfite sequencing for assessing epigenetic heterogeneity. Nat Methods 2014;8:817–820. 10.1038/nmeth.3035 PMC4117646 25042786

[dmy021C186] Smallwood SA, Tomizawa S, Krueger F, Ruf N, Carli N, Segonds-Pichon A, Sato S, Hata K, Andrews SR, Kelsey G Dynamic CpG island methylation landscape in oocytes and preimplantation embryos. Nat Genet 2011;8:811–814. 10.1038/ng.864 PMC3146050 21706000

[dmy021C187] Smith ZD, Chan MM, Humm KC, Karnik R, Mekhoubad S, Regev A, Eggan K, Meissner A DNA methylation dynamics of the human preimplantation embryo. Nature 2014;7511:611–615. 10.1038/nature13581 PMC4178976 25079558

[dmy021C188] Smith ZD, Chan MM, Mikkelsen TS, Gu H, Gnirke A, Regev A, Meissner A A unique regulatory phase of DNA methylation in the early mammalian embryo. Nature 2012;7394:339–344. 10.1038/nature10960 PMC3331945 22456710

[dmy021C189] Smith ZD, Meissner A DNA methylation: roles in mammalian development. Nat Rev Genet 2013;3:204–220. 10.1038/nrg3354 23400093

[dmy021C190] Stewart KR, Veselovska L, Kim J, Huang J, Saadeh H, Tomizawa S, Smallwood SA, Chen T, Kelsey G Dynamic changes in histone modifications precede de novo DNA methylation in oocytes. Genes Dev 2015;23:2449–2462. 10.1101/gad.271353.115 PMC4691949 26584620

[dmy021C191] Surani MA, Barton SC, Norris ML Development of reconstituted mouse eggs suggests imprinting of the genome during gametogenesis. Nature 1984;5959:548–550. 10.1038/308548a0 6709062

[dmy021C192] Tachibana M, Sugimoto K, Nozaki M, Ueda J, Ohta T, Ohki M, Fukuda M, Takeda N, Niida H, Kato H et al G9a histone methyltransferase plays a dominant role in euchromatic histone H3 lysine 9 methylation and is essential for early embryogenesis. Genes Dev 2002;14:1779–1791. 10.1101/gad.989402 PMC186403 12130538

[dmy021C193] Tachibana M, Ueda J, Fukuda M, Takeda N, Ohta T, Iwanari H, Sakihama T, Kodama T, Hamakubo T, Shinkai Y Histone methyltransferases G9a and GLP form heteromeric complexes and are both crucial for methylation of euchromatin at H3-K9. Genes Dev 2005;7:815–826. 10.1101/gad.1284005 PMC1074319 15774718

[dmy021C194] Takagi N, Sasaki M Preferential inactivation of the paternally derived X chromosome in the extraembryonic membranes of the mouse. Nature 1975;5519:640–642. 10.1038/256640a0 1152998

[dmy021C195] Tang Q, Chen Y, Wu W, Ding H, Xia Y, Chen D, Wang X Idiopathic male infertility and polymorphisms in the DNA methyltransferase genes involved in epigenetic marking. Sci Rep 2017;1:11219. 10.1038/s41598-017-11636-9 PMC5593912 28894282

[dmy021C196] Tomizawa S, Nowacka-Woszuk J, Kelsey G DNA methylation establishment during oocyte growth: mechanisms and significance. Int J Dev Biol 2012;10-12:867–875. 10.1387/ijdb.120152gk 23417409

[dmy021C197] Tomizawa S, Sasaki H Genomic imprinting and its relevance to congenital disease, infertility, molar pregnancy and induced pluripotent stem cell. J Hum Genet 2012;2:84–91. 10.1038/jhg.2011.151 22237588

[dmy021C198] Torregrosa N, Dominguez-Fandos D, Camejo MI, Shirley CR, Meistrich ML, Ballesca JL, Oliva R Protamine 2 precursors, protamine 1/protamine 2 ratio, DNA integrity and other sperm parameters in infertile patients. Hum Reprod 2006;8:2084–2089. 10.1093/humrep/del114 16632464

[dmy021C199] Torres IO, Fujimori DG Functional coupling between writers, erasers and readers of histone and DNA methylation. Curr Opin Struct Biol 2015;35:68–75. 26496625 10.1016/j.sbi.2015.09.007 PMC4688207

[dmy021C200] Urdinguio RG, Bayon GF, Dmitrijeva M, Torano EG, Bravo C, Fraga MF, Bassas L, Larriba S, Fernandez AF Aberrant DNA methylation patterns of spermatozoa in men with unexplained infertility. Hum Reprod 2015;5:1014–1028. 10.1093/humrep/dev053 25753583

[dmy021C201] Veselovska L, Smallwood SA, Saadeh H, Stewart KR, Krueger F, Maupetit-Mehouas S, Arnaud P, Tomizawa S, Andrews S, Kelsey G Deep sequencing and de novo assembly of the mouse oocyte transcriptome define the contribution of transcription to the DNA methylation landscape. Genome Biol 2015;16:209. 26408185 10.1186/s13059-015-0769-z PMC4582738

[dmy021C202] Wadhwa PD, Buss C, Entringer S, Swanson JM Developmental origins of health and disease: brief history of the approach and current focus on epigenetic mechanisms. Semin Reprod Med 2009;5:358–368. 10.1055/s-0029-1237424 PMC2862635 19711246

[dmy021C203] Wang C, Lee JE, Lai B, Macfarlan TS, Xu S, Zhuang L, Liu C, Peng W, Ge K Enhancer priming by H3K4 methyltransferase MLL4 controls cell fate transition. Proc Natl Acad Sci U S A 2016;42:11871–11876. 10.1073/pnas.1606857113 PMC5081576 27698142

[dmy021C204] Wang X, Moore SC, Laszckzak M, Ausio J Acetylation increases the alpha-helical content of the histone tails of the nucleosome. J Biol Chem 2000;45:35013–35020. 10.1074/jbc.M004998200 10938086

[dmy021C205] Waterland RA, Jirtle RL Transposable elements: targets for early nutritional effects on epigenetic gene regulation. Mol Cell Biol 2003;15:5293–5300. 10.1128/MCB.23.15.5293-5300.2003 PMC165709 12861015

[dmy021C206] Weimar CH, Post Uiterweer ED, Teklenburg G, Heijnen CJ, Macklon NS In-vitro model systems for the study of human embryo-endometrium interactions. Reprod Biomed Online 2013;5:461–476. 10.1016/j.rbmo.2013.08.002 24055530

[dmy021C207] White CR, Denomme MM, Tekpetey FR, Feyles V, Power SG, Mann MR High frequency of imprinted methylation errors in human preimplantation embryos. Sci Rep 2015;5:17311. 26626153 10.1038/srep17311 PMC4667293

[dmy021C208] Wright SJ Sperm nuclear activation during fertilization. Curr Top Dev Biol 1999;46:133–178. 10417879 10.1016/s0070-2153(08)60328-2

[dmy021C209] Wu J, Huang B, Chen H, Yin Q, Liu Y, Xiang Y, Zhang B, Liu B, Wang Q, Xia W et al The landscape of accessible chromatin in mammalian preimplantation embryos. Nature 2016;7609:652–657. 10.1038/nature18606 27309802

[dmy021C210] Wu J, Xu J, Liu B, Yao G, Wang P, Lin Z, Huang B, Wang X, Li T, Shi S et al Chromatin analysis in human early development reveals epigenetic transition during ZGA. Nature 2018. 10.1038/s41586-018-0080-8 29720659

[dmy021C211] Wu X, Zhang Y TET-mediated active DNA demethylation: mechanism, function and beyond. Nat Rev Genet 2017;9:517–534. 10.1038/nrg.2017.33 28555658

[dmy021C212] Xie W, Schultz MD, Lister R, Hou Z, Rajagopal N, Ray P, Whitaker JW, Tian S, Hawkins RD, Leung D et al Epigenomic analysis of multilineage differentiation of human embryonic stem cells. Cell 2013;5:1134–1148. 10.1016/j.cell.2013.04.022 PMC3786220 23664764

[dmy021C213] Xue L, Cai JY, Ma J, Huang Z, Guo MX, Fu LZ, Shi YB, Li WX Global expression profiling reveals genetic programs underlying the developmental divergence between mouse and human embryogenesis. BMC Genomics 2013;14:568. 23961710 10.1186/1471-2164-14-568 PMC3924405

[dmy021C214] Yan L, Yang M, Guo H, Yang L, Wu J, Li R, Liu P, Lian Y, Zheng X, Yan J et al Single-cell RNA-Seq profiling of human preimplantation embryos and embryonic stem cells. Nat Struct Mol Biol 2013;9:1131–1139. 10.1038/nsmb.2660 23934149

[dmy021C215] Yang X, Hu B, Hou Y, Qiao Y, Wang R, Chen Y, Qian Y, Feng S, Chen J, Liu C et al Silencing of developmental genes by H3K27me3 and DNA methylation reflects the discrepant plasticity of embryonic and extraembryonic lineages. Cell Res 2018. 10.1038/s41422-018-0010-1 PMC5951795 29463899

[dmy021C216] Yao TP, Oh SP, Fuchs M, Zhou ND, Ch’ng LE, Newsome D, Bronson RT, Li E, Livingston DM, Eckner R Gene dosage-dependent embryonic development and proliferation defects in mice lacking the transcriptional integrator p300. Cell 1998;3:361–372. 10.1016/s0092-8674(00)81165-4 9590171

[dmy021C217] Yu B, Dong X, Gravina S, Kartal O, Schimmel T, Cohen J, Tortoriello D, Zody R, Hawkins RD, Vijg J Genome-wide, single-cell dna methylomics reveals increased non-CpG methylation during human oocyte maturation. Stem Cell Reports 2017;1:397–407. 10.1016/j.stemcr.2017.05.026 PMC5511109 28648898

[dmy021C218] Yuen RK, Jiang R, Penaherrera MS, McFadden DE, Robinson WP Genome-wide mapping of imprinted differentially methylated regions by DNA methylation profiling of human placentas from triploidies. Epigenetics Chromatin 2011;1:10. 10.1186/1756-8935-4-10 PMC3154142 21749726

[dmy021C219] Zenk F, Loeser E, Schiavo R, Kilpert F, Bogdanovic O, Iovino N Germ line-inherited H3K27me3 restricts enhancer function during maternal-to-zygotic transition. Science 2017;6347:212–216. 10.1126/science.aam5339 28706074

[dmy021C220] Zhang T, Cooper S, Brockdorff N The interplay of histone modifications – writers that read. EMBO Rep 2015;11:1467–1481. 10.15252/embr.201540945 PMC4641500 26474904

[dmy021C221] Zhang Y, Xiang Y, Yin Q, Du Z, Peng X, Wang Q, Fidalgo M, Xia W, Li Y, Zhao ZA et al Dynamic epigenomic landscapes during early lineage specification in mouse embryos. Nat Genet 2018;1:96–105. 10.1038/s41588-017-0003-x 29203909

[dmy021C222] Zhang B, Zheng H, Huang B, Li W, Xiang Y, Peng X, Ming J, Wu X, Zhang Y, Xu Q et al Allelic reprogramming of the histone modification H3K4me3 in early mammalian development. Nature 2016;7621:553–557. 10.1038/nature19361 27626382

[dmy021C223] Zhao Y, Garcia BA Comprehensive catalog of currently documented histone modifications. Cold Spring Harb Perspect Biol 2015;9:a025064. 10.1101/cshperspect.a025064 PMC4563710 26330523

[dmy021C224] Zheng H, Huang B, Zhang B, Xiang Y, Du Z, Xu Q, Li Y, Wang Q, Ma J, Peng X et al Resetting epigenetic memory by reprogramming of histone modifications in mammals. Mol Cell 2016;6:1066–1079. 10.1016/j.molcel.2016.08.032 27635762

[dmy021C225] Zheng HY, Tang Y, Niu J, Li P, Ye DS, Chen X, Shi XY, Li L, Chen SL Aberrant DNA methylation of imprinted loci in human spontaneous abortions after assisted reproduction techniques and natural conception. Hum Reprod 2013;1:265–273. 10.1093/humrep/des358 23042795

[dmy021C226] Zhu P, Guo H, Ren Y, Hou Y, Dong J, Li R, Lian Y, Fan X, Hu B, Gao Y et al Single-cell DNA methylome sequencing of human preimplantation embryos. Nat Genet 2018;1:12–19. 10.1038/s41588-017-0007-6 29255258

[dmy021C227] Zuccotti M, Piccinelli A, Giorgi Rossi P, Garagna S, Redi CA Chromatin organization during mouse oocyte growth. Mol Reprod Dev 1995;4:479–485. 10.1002/mrd.1080410410 7576615

[dmy021C228] Zylicz JJ, Dietmann S, Gunesdogan U, Hackett JA, Cougot D, Lee C, Surani MA Chromatin dynamics and the role of G9a in gene regulation and enhancer silencing during early mouse development. Elife 2015;4:e09571. 26551560 10.7554/eLife.09571 PMC4729692

